# Hepatitis C Virus Infection Epidemiology among People Who Inject Drugs in Europe: A Systematic Review of Data for Scaling Up Treatment and Prevention

**DOI:** 10.1371/journal.pone.0103345

**Published:** 2014-07-28

**Authors:** Lucas Wiessing, Marica Ferri, Bart Grady, Maria Kantzanou, Ida Sperle, Katelyn J. Cullen, EMCDDA DRID group, Angelos Hatzakis, Maria Prins, Peter Vickerman, Jeffrey V. Lazarus, Vivian D. Hope, Catharina Matheï

**Affiliations:** 1 European Monitoring Centre for Drugs and Drug Addiction (EMCDDA), Lisbon, Portugal; 2 Cluster Infectious Diseases, Department of Research, Public Health Service, Amsterdam, The Netherlands; 3 Center for Infection and Immunity Amsterdam (CINIMA), Academic Medical Center, Amsterdam, The Netherlands; 4 National Reference Centre for Retroviruses, Laboratory of Hygiene, Epidemiology and Medical Statistics, University of Athens Medical School, Athens, Greece; 5 Copenhagen HIV Programme (CHIP), Rigshospitalet, University of Copenhagen, Copenhagen, Denmark; 6 HIV & STI Department, Centre for Infectious Disease Surveillance and Control, Public Health England, London, United Kingdom; 7 London School of Hygiene and Tropical Medicine (LSHTM), London, United Kingdom; 8 School of Social and Community Medicine, University of Bristol, Bristol, United Kingdom; 9 Department of Public Health and Primary Care, KULeuven, Leuven, Belgium; Centers for Disease Control and Prevention, United States of America

## Abstract

**Background:**

People who inject drugs (PWID) are a key population affected by hepatitis C virus (HCV). Treatment options are improving and may enhance prevention; however access for PWID may be poor. The availability in the literature of information on seven main topic areas (incidence, chronicity, genotypes, HIV co-infection, diagnosis and treatment uptake, and burden of disease) to guide HCV treatment and prevention scale-up for PWID in the 27 countries of the European Union is systematically reviewed.

**Methods and Findings:**

We searched MEDLINE, EMBASE and Cochrane Library for publications between 1 January 2000 and 31 December 2012, with a search strategy of general keywords regarding viral hepatitis, substance abuse and geographic scope, as well as topic-specific keywords. Additional articles were found through structured email consultations with a large European expert network. Data availability was highly variable and important limitations existed in comparability and representativeness. Nine of 27 countries had data on HCV incidence among PWID, which was often high (2.7-66/100 person-years, median 13, Interquartile range (IQR) 8.7–28). Most common HCV genotypes were G1 and G3; however, G4 may be increasing, while the proportion of traditionally ‘difficult to treat’ genotypes (G1+G4) showed large variation (median 53, IQR 43–62). Twelve countries reported on HCV chronicity (median 72, IQR 64–81) and 22 on HIV prevalence in HCV-infected PWID (median 3.9%, IQR 0.2–28). Undiagnosed infection, assessed in five countries, was high (median 49%, IQR 38–64), while of those diagnosed, the proportion entering treatment was low (median 9.5%, IQR 3.5–15). Burden of disease, where assessed, was high and will rise in the next decade.

**Conclusion:**

Key data on HCV epidemiology, care and disease burden among PWID in Europe are sparse but suggest many undiagnosed infections and poor treatment uptake. Stronger efforts are needed to improve data availability to guide an increase in HCV treatment among PWID.

## Introduction

Chronic infection with the hepatitis C virus (HCV) affects about 160 million people worldwide [1], [2]. In developed countries, iatrogenic transmission of this blood-borne virus has been substantially reduced and people who inject drugs (PWID), or those who have done so in the past, are now the main group affected [3]–[6].

HCV infection is a serious public health problem as chronically infected individuals are at risk for long-term sequelae, including liver cirrhosis and hepatocellular carcinoma [7]. Indeed, in Europe, HCV is a leading cause of cirrhosis and primary liver cancer [8]. Since 2001, effective treatment with pegylated interferon and ribavirin has been available. In recent years, there have been advances in treatment with the development of direct acting antiviral (DAA) therapy [9]. PWID in many countries still have limited access to HCV treatment, despite multiple studies providing evidence that this population can be successfully treated [10]–[17] only 1–6% of HCV-infected current and former PWID in the United States, Canada, and Australia were treated [14]–[16], [18]–[20]. The low uptake of treatment among PWID is due to both physician and patient-associated factors. Firstly, physicians’ concerns about adherence, other co-morbidities including HIV co-infection, treatment side-effects and the potential for re-infection may lead to treatment being withheld [5], [10], [21], [22]. Secondly, poverty, psychiatric co-morbidities, poor social support and stigma are common among PWID and may result in HCV treatment not being viewed as a priority for them [23]–[25]. Other barriers may relate to educational level, problems with accessing diagnostic tests (e.g. in non-urban regions or when access to primary care is difficult), and entering specialist referral pathways [26], [27].

Treatment is likely to have a synergistic impact on HCV prevention efforts. Modelling studies suggest that antiviral treatment could play an important, and cost-effective, role in preventing HCV in PWID by reducing the number at risk of transmitting HCV [28]–[31].

In Europe, many countries have implemented harm reduction programmes [32], [33] as well as health insurance systems to cover treatment costs of PWID (several including DAA). Therefore, access to HCV treatment should be feasible [34], [35] and recent European clinical guidelines state it must be considered for PWID [36]. Although national treatment guidelines have varied substantially and have often been highly restrictive with regard to PWID [37], [38], the experience in some European countries has shown that it is possible to expand HCV diagnosis, prevention and treatment of PWID [6], [34], [39]–[42]. Key data elements to inform HCV treatment scale-up for PWID cover epidemiological data on the prevalence, dynamics and characteristics of the epidemic, estimates of future burden of disease and associated healthcare needs [39], [43]–[47].

To assess data availability for informing a potential future scale up of HCV treatment (including ‘treatment for prevention’) among PWID in Europe, we performed a systematic review of the literature published between 2000–2012, covering the epidemiology of HCV infection, treatment uptake and estimates of the future burden of disease among PWID in European countries (the EU 27) to complement existing routinely collated data (for example on antibody prevalence and harm reduction service provision as collected by EMCDDA – see Table S2 in Web-appendix S1) [4], [44], [48], [49]. Most of these data might be equally important to informing overall HCV prevention policies. (Further detail on the rationale and importance of these data for HCV treatment and prevention policies is given in Table 1 and Discussion, while Table 2 summarises what this study adds to current knowledge.).

**Table 1:** Data items reviewed and their rationale for HCV policy.

1. HCV incidence in PWID: complement available prevalence data by giving an estimate for level of recent infections, a direct measure of (treatment for) prevention effectiveness as well as being important for future burden estimates.
2. Chronicity of infections: allow interpreting antibody prevalence data in terms of current and projected future prevalence of infection and treatment need.
3. Genotypes: predictor of current treatment outcomes, Genotypes 1 and 4 are traditionally hard to treat. New treatments may overcome this problem but are not yet implemented at scale and there are important costs issues.
4. HIV co-infection: predictor of current treatment outcomes and mortality (new treatments may overcome this: see previous point).
5. Undiagnosed proportion: extent of under-diagnosis and linkage to care and treatment.
6. Treatment entry: measure of treatment coverage and accessibility.
7. Burden of disease: projections of future costs to the health care system and wider society, important when considering investment into treatment

**Table 2:** What is known and what this study adds.

1. More specialised reviews have been carried out [43], [50]–[56], but no systematic review has provided a comprehensive overview [57], across multiple topic areas, of available data to inform hepatitis C treatment scale-up (including prevention) among PWID in a large region of the world.
2. Our review found that the majority of available studies published between 2000–2012 focused on HCV prevalence, treatment and on the genotype characterisation of patients with HCV, while very few investigated the burden of disease. In some of the topic areas data was scarce, in particular for recent years (2006–2012).
3. Incidence of HCV infection in PWID in the EU varies greatly, but can be as high as 66/100 person years (PY). Chronicity rates vary both above and under the expected 75% [56]. Genotypes 1 and 3 predominate among PWID, but 4 appears to be increasing, while the proportion of ‘difficult to treat’ genotypes (1+4) shows large variation (17–91%, median 53%). The prevalence of HIV co-infection in HCV-infected PWID varies widely (0–70%, median 3.9%), correlating closely with but generally higher than overall HIV prevalence among PWIDs.
4. Half of the chronically infected PWID were unaware of their infection, and, of those diagnosed, only one in ten entered treatment for Hepatitis C.
5. This study highlights major information gaps regarding epidemiology, diagnosis, treatment entry and burden of disease of hepatitis C infection in PWID in most European Union countries, potentially hampering HCV treatment scale up.

Our overall research question was: ‘What data is available in European Union countries to inform a potential scale-up of HCV treatment (for prevention) among PWID?’ This was operationalised into five specific questions, covering seven topic areas, all limited to HCV infection among PWID in Europe:

1. What is the incidence of infection?
2. What proportion of infections become chronic?
3. What are infection characteristics, in terms of genotypes and HIV co-infection?
4. What proportions of infected are undiagnosed and, of those diagnosed, enter antiviral treatment?
5. What estimates exist of the future burden of disease?

## Methods

Seven separate systematic reviews of the literature were performed, covering HCV incidence, chronicity rates, genotype distribution, HIV co-infection, undiagnosed chronic hepatitis C (CHC) cases, HCV treatment uptake, and burden of disease. Study references were identified through searches of MEDLINE, EMBASE and the Cochrane Library databases for articles published in any language between 1 January 2000 and 31 December 2012. A standard search strategy was agreed (Web-appendix S1) with general keywords regarding viral hepatitis, substance abuse and geographic scope (Table 3), as well as seven search strings with topic area-specific keywords. Additional articles were found through structured email consultations with a large European expert network (on substance use and infectious diseases, including viral hepatitis (see Acknowledgments)) covering each of the 27 countries (Table 4). The protocol was consistent with the PRISMA criteria [58]. Search results per topic area were screened for relevance independently by two researchers on the basis of title and abstract and results compared, retaining articles in case of doubt. Duplicates between the three databases were removed. The remaining articles were retrieved and evaluated independently by two researchers on the basis of the full article text using agreed selection criteria across all seven topic areas. Studies were included for the 27 EU member states. Non-English articles were evaluated with the help of native speakers, country experts and online translation services (Google Translate and BabelFish) and if necessary and possible by contacting the authors. Additional inclusion criteria were reporting data collected since 1990 (except for Burden of disease, due to the long-term perspective of modelling projections). Quality criteria for inclusion (Web-appendix S1) were to have a clearly defined study population of PWID only (having ever injected drugs) or data provided for PWID separately, consistency and clarity of the data reported and an unselected sample of PWID with regard to gender or HBV/HIV co-infection (except for Burden of disease, where due to the small number of studies found, two studies based on HIV co-infected samples were included). Sample sizes below n = 10 were excluded except if they provided the only data for a country on a topic area. For articles excluded in this phase the reasons for this were noted. Data were extracted from the remaining articles into tables for each of the topic areas (Tables S3–S10 in Web-appendix S2) and the reference lists were checked for further studies. Multiple publications for one study were consolidated and treated as one entry, in order to maximise information available per study. Data were presented untransformed (Incidence, Burden of disease) or, where possible, pooled weighted prevalences (median, average, range in %) were calculated per country (Chronicity, Genotypes, Co-infection, Diagnosis, Treatment entry) and study type/setting (Incidence, Chronicity, Diagnosis, Treatment entry). Data availability per country was crudely assessed by the number of (out of seven) topic areas where data were available (Table 4). For Genotypes the sum of the proportions of ‘difficult to treat’ genotypes (1 and 4) [36], [59], [60] is presented (Table 4). For Co-infection the correlation was assessed with HIV prevalence. Overall, a meta-analysis was considered outside the scope of this review, although for one topic (Chronicity) a limited analysis was performed (Web-appendix S3).

**Table 3:** Search terms used.

General terms across all topic areas: (“Substance Abuse, Intravenous”[Mesh] OR “IDU” OR “IDUs” OR “PWID” OR “IVDU” OR “IVDUs” OR “intravenous drug” OR “injecting drug” OR “intravenous substance” OR “Injection drug” OR “inject drugs”) AND (“Hepatitis C”[Mesh] OR “hepatitis C” OR “HCV”) as well as country specific terms.
Additional search strings per topic area: 1) Incidence: “incidence”, 2) Chronicity: HCV-RNA[All Fields] OR (“genotype”[MeSH Terms] OR “genotype”[All Fields]) OR persistence[All Fields] OR (“viraemia”[All Fields] OR “viremia”[MeSH Terms] OR “viremia”[All Fields]), 3) Genotype: “genotype”[tiab] OR “subtype”[tiab] OR “molecular epidemiology” [tiab], 4) Co-infection: HIV or “HIV”[Mesh] or “hiv*”, 5) Diagnosis: “test”[tiab] OR “prevalence”[tiab] OR “proportion”[tiab] OR “referral”[tiab] OR “trend”[tiab] OR “screening”[tiab] OR “diagnostics”[tiab] OR “surveillance”[tiab] OR “unidentified”[tiab] OR “diagnosis”[tiab] OR “undiagnosed”[tiab], 6) Treatment: “antiviral”[tiab] OR “treatment”[tiab] OR “therapeutics”[tiab] OR “access to treatment”[tiab], 7) Burden of Disease: Cost-effectiv* [tiab] OR burden [tiab] OR daly [tiab] OR qaly[tiab] OR morbidity [tiab] OR mortality[tiab] OR “Cost of Illness”[Mesh] OR illness cost*[tiab] OR incremental cost-effectiveness ratio [tiab] OR Cost-Benefit Analysis [Mesh].
Country specific terms: Web-appendix S1.

**Table 4:** HCV infection among people who inject drugs in the European Union - availability by country of key data to scale up antiviral treatment (figures that include data since 2006 (inclusive) are shown in **bold** and figures not included in the final analyses are in square brackets []).

	Incidence /100PY of primary infection of HCV in PWID [reinfection]	Chronicity: weighted mean prevalence of HCV RNA (%) among antibody positive PWID (range:number of studies:overall sample size)	Genotype 1 or 4: weighted mean prevalence of the sum of HCV genotypes 1 and 4 among PWID (%) (range:number of studies:overall sample size: G1/G2/G3/G4)@	Co-infection HIV: weighted mean HIV prevalence among HCV antibody positive PWID (%) (range: number of studies: overall sample size)	Diagnosis: weighted mean proportion HCV positive PWID (ab or RNA) who were undiagnosed (%)(range:number of studies:overall sample size)	Treatment entry: weighted mean proportion HCV-infected PWID entering antiviral treatment, (%) (range: number of studies: overall sample size)	Burden of disease from HCV infection in PWID (mortality, liver disease)	Topic areas of this review covered	Written viral hepatitis policy exists#
Austria	NA	83 (NA:1∶139) [acute 0.0 (NA:1∶3)]	53 (NA:1∶75: 48/1.3/40/5.3)	**0.7 $ (NA:1∶309)**	NA	**[int-nonclin 12 (NA:1∶141)]**	NA	4/7	Yes
Belgium	NA	**78 $ (67–95∶3:627)**	50 $ (47–52∶2:271∶41/1.7/47/9.1)	**Multisite 4.9 (2.1–5.9∶2:485)**	NA	NA	NA	3/7	No
Bulgaria	NA	**78 (NA:1∶147)**	**61 (35–65∶2:133∶61/0.0/38/0.0)**	**Sofia 3.9 (NA:1∶955)**	NA	NA	NA	3/7	No
Cyprus	NA	**70 (NA:1∶20)**	**43 (NA:1∶14: 43/0.0/57/0.0)**	**0.0 $ (NA:1∶40)**	NA	NA	NA	3/7	No
Czech Republic	Multisite 11 $ (2002–2005), Karvina: 15 (1998–2001)	**69 $ (59–71∶2:546)**	**79 (76–91∶2:275∶79/0.4/20/0.0)**	**Multisite 0.0 $ (0.0–0.0∶4:873)**	NA	NA	NA	4/7	No&
Denmark	Nyborg prison: 25 (1996–97)	**67 $ (53–68∶3:3615)**	NA	**Nyborg prison 0.0 (0.0–0.0∶2:268)**	**46 $ (NA:1∶9463) (ab or RNA)**	NA	**Observed mortality $: MR 12 / 100 (1995–2006)**	5/7	Yes
Estonia	NA	NA	63 (NA:1∶35: 63/0.0/37/0.0)	62 (NA:1∶374)	NA	NA	NA	1/7	No
Finland	Helsinki, Tampere and Turku: 31 (2000–2002)	NA	NA	NA	NA	NA	NA	1/7	No
France	Northeast: 9.1 (1999–2000)	81 $ (73–85∶5:785)	55 (52–60∶4:1174∶46/2.5/37/9.1)	Multisite 18 (2.4–68∶4:1444)	30 $ (NA:1∶91) (ab)	obs-nonclin 18 $ (1.8–19∶3:2453) [obs-clin 25 $ (8.8–28∶3:1421), int-nonclin 29 (17–38∶2:387)]	NA	6/7	Yes
Germany	[Re-infection: Munich: 0–4.1 (1997–2000)]	NA	61 (54–65∶3:300∶63/3.8/31/2.6)	**Multisite 7.1 (7.1–7.2∶3:681)**	NA	**obs-nonclin 8.6 $ (NA:1∶301)** [int-clin 47 (NA:1∶106)]	NA	4/7	No
Greece	NA	NA	**36 $ (32–46∶7:1774∶24/2.8/61/11)**	**Multisite 0.8 $£ (0.0–1.7∶12:8,626)**	NA	**[obs-clin 59 (NA:1∶305)]**	NA	3/7	Yes&
Hungary	NA	NA	NA	**Multisite 0.0 (0.0–0.0∶2:396)**	NA	**obs-nonclin 0.9 (NA:1∶234) [obs-clin 29 (NA:1∶123)]**	NA	2/7	No
Ireland	Dublin: 25 (2001), 66 (1992–1999)	63 (62–81∶2:532)	50 (NA:1∶299: 49/2.0/49/0.7)	NA	NA	**obs-nonclin 10 (NA:1∶29)**, [obs-nonclin self-ab+ ever-Tx 4.5 (NA:1∶22) int-nonclin 7.7 (NA:1∶26)]	NA	5/7	No
Italy	NA	97 $ (1∶406) **[acute 56 (NA:1∶71)]**	**58 (54–61∶3:427∶45/3.3/38/13)**	**Multisite 16 (5.2–23∶5:3,177)**	NA	[int-nonclin 31 (NA:1∶169)]	NA	4/7	No
Latvia	NA	NA	NA	**Multisite 30 (27–35∶3:1,080)**	NA	**[obs-nonclin self-ab+ ever-Tx 4.8 (NA:1∶314)]**	NA	2/7	No
Lithuania	NA	NA	**17 (NA:1∶12: 17/8.3/75/0.0)**	**Vilnius 8.1 (NA:1∶400)**	NA	NA	NA	2/7	No
Luxembourg	NA	NA		3.0 $ (NA:1∶202)	NA	NA	NA	1/7	No
Malta	NA	NA	NA	NA	NA	NA	NA	0/7	No
Netherlands	Amsterdam: 6.8 (1985–2005) **[Re-infection: Amsterdam 3.4 (2005–2010)]**	67 (NA:1∶106)	66 (62–71∶2:128∶53/6.0/32/9.0)	Amsterdam 30 (NA:1∶952)	NA	**[int-nonclin 33 (23–48∶2:184)]**	**Observed mortality: MR 2.3 /100 (1985–2009) Modelled: cirrhosis and HCC: 36% increase in liver disease burden 2011–2025, Amsterdam**	5/7	No
Poland	NA	NA	**44 (NA:1∶23: 35/0.0/57/8.7)**	**Multisite 31 (4–55∶9:1109)**	**56 (24–65∶3:562) (RNA)**	NA	NA	3/7	No
Portugal	NA	NA	**79 (NA:1∶52: 62/0.0/21/17)**	Tires 48 (NA:1∶45)	NA	NA	NA	2/7	No&
Romania	NA	NA	**85 (NA:1∶26: 73/0.0/7.7/12)**	**Bucharest 0.0 £ (NA:1∶45)**	NA	NA	NA	2/7	Yes&
Slovakia	NA	NA			NA	NA	NA	0/7	No
Slovenia	NA	NA	**40 $ (NA:1∶516: 39/1.6/58/1.7)**	**0.8 $ (0–1.6∶4:1063)**	NA	NA	NA	2/7	No&
Spain	Madrid, Barcelona and Seville: 28 (2001–2003)	NA	69 (68–72∶2:256∶54/2.3/27/16)	**Multisite 50 (30–70∶10:9,646)**	**25 (NA:1∶545) (ab/RNA NA)**	[obs-clin 54 (NA:1∶494)]	**Observed mortality: MR 2.1–2.4 /100 (1990–2002, 1997–2008)**	6/7	No
Sweden	Malmö: 38 (1997–2005), 26 (1990–93)	**77 (NA:1∶268)**	**38 (NA:1∶206: 36/8.7/34/0.9)**	NA	NA	NA	NA	3/7	No
UK	**16 studies: 2.7 – 42 (2001–2009) mean 14 [Re-infection: Glasgow, 6.9, (1993–2007)]**	**70 (60–71∶3:2581)**	**50 (44–58∶6:492∶49/5.7/42/0.8)**	**Multisite 2.2 (0–8∶11:26,111) $**	**59 (49–76∶4:1696) (ab or RNA) [58 (NA:1:NA) (RNA)]**	**[**obs-clin 28 (NA:1∶179), **obs non-clin self-ab+ ever-Tx 37 (NA:1∶414), int non-clin 23 (22–25∶2:559)]**	**Modelled: 56% increase of cirrhosis, 64% of moderate liver disease from 2010–2025, Glasgow**	7/7	Yes

Data are shown with two significant digits. # Source: The Global Viral Hepatitis Report, WHO 2013. Country response to the question: “In your country, is there a written national strategy or plan that focuses exclusively or primarily on the prevention and control of viral hepatitis?”. EMCDDA DRID Group respondents were asked to review and if necessary adjust (indicated by &). ab = antibody, clin = clinical, int = intervention study, nonclin = non-clinical, obs = observational study, NA = not available, RCT = Randomised Controlled Trial, RNA = ribonucleic acid, self = self-reported, Tx = treated, $ = includes studies with national coverage. £ In 2010 an HIV outbreak occurred among PWID in Greece and Romania, therefore the here presented figures are likely incorrect and co-infection is much higher. @ Genotypes 1 and 4 are harder to treat with conventional treatments, the total here thus gives the proportion of ‘hard to treat’ cases; “G1/G2/G3/G4” denotes percentages of genotypes 1 to 4 separately; small discrepancies between the sum of genotypes 1 and 4 and separate percentages of genotypes 1 and 4 are due to missing values. For full detail on data see Web-appendix S2.

## Results

**Figure 1:**
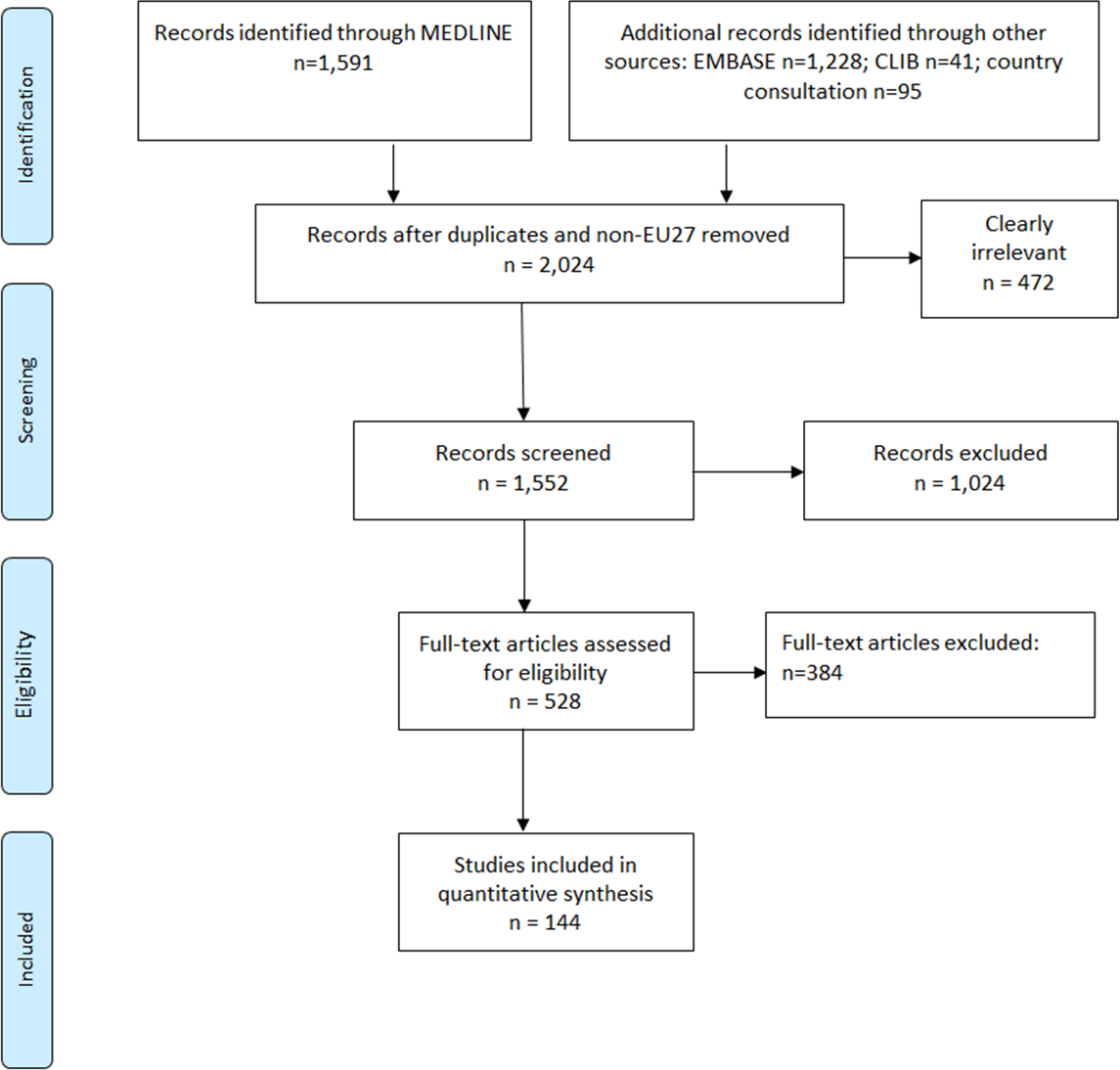
Flow diagram of study selection aggregated over the seven topic areas reviewed.

Overall the systematic search retrieved 2,955 references to publications. After removal of duplicates and clearly irrelevant references, we screened 1,552 references on the basis of title and abstract. 528 articles were retrieved and obtained in full text, of which 144 were included in the quantitative synthesis. The step-wise description of selection and inclusion of studies is depicted in a flowchart in Figure 1. Followed below are detailed study questions, main findings and results for each of the seven topic areas reviewed.

### Incidence

#### Study questions

What is the incidence of HCV infection among PWID in Europe and how has this been measured? How does incidence vary between countries?

#### Main findings

**Figure 2:**
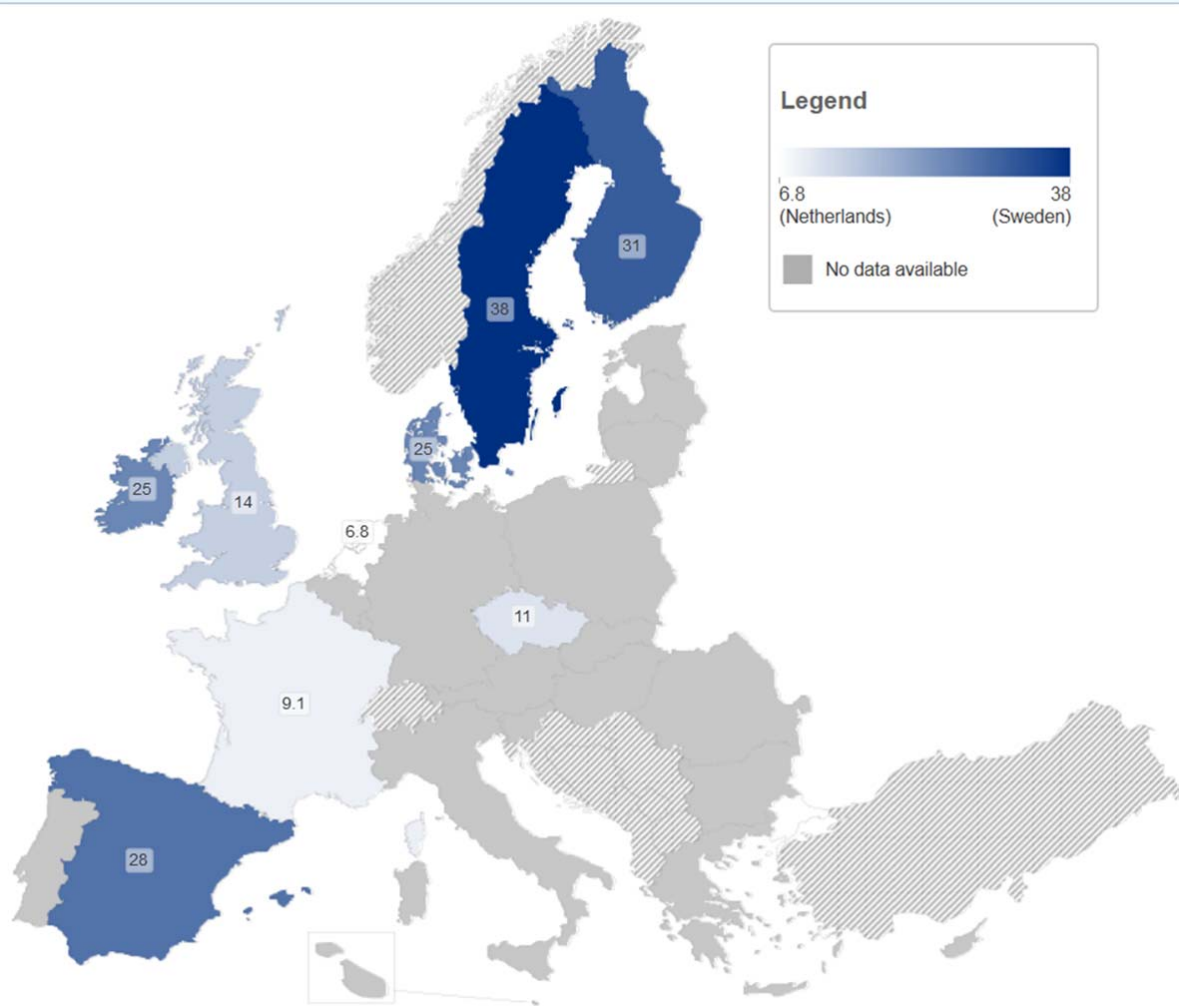
Incidence of HCV infection in PWID (per 100 person years).

Data are sparse across Europe and are not easily comparable. The data suggests that incidence is highly variable in the populations studied (Figure 2).

#### Studies included

Studies were included that had directly measured incidence of hepatitis C infection in PWID, by using one of the following approaches: 1) a cohort or follow-up study 2) detection of HCV RNA in the absence of anti-HCV in a cross-sectional study 3) assessing anti-HCV avidity in a cross-sectional study. Studies that had indirectly estimated incidence from HCV prevalence data, for example using force of infection calculations, were excluded. 27 studies, from nine countries, reported the incidence of primary HCV infection (Czech Republic, Denmark, Ireland, Finland, France, Netherlands, Sweden, Spain, UK; Table 4). Four countries had undertaken more than one (Czech Republic, Ireland, Sweden, UK); most studies had been undertaken in UK (n = 16, 59%). Three studies measured re-infection (Germany, Netherlands, UK; Table 4) (Table S3 in Web-appendix S2). [61]–[89].

#### Study design

Incidence of primary HCV infection had been measured in 17 follow-up studies, and in ten cross-sectional studies (nine used HCV RNA in the absence of anti-HCV and one anti-HCV avidity to estimate HCV incidence).

#### Population

For primary HCV infection, three studies recruited PWID from community settings, 19 through health services (including needle and syringe programmes) and three from both settings. Two were in custodial settings. The re-infection studies were in clinical settings.

#### Findings

The number of PWID at risk of primary infection in studies undertaken outside of custodial settings ranged from 27 (Spain, ‘new PWID’: injecting less than 2 years) [68] to 2,532 (UK, ‘ever PWID’: having ever injected drugs) [82]; (median:168 people; mean 424). The measured incidence of primary HCV infection varied from 2.7–3.2/100PY in one UK study (ever PWID) [82] to 66/100PY in a study from Ireland (ever PWID) [66]. The median incidence was 13/100PY (Interquartile range (IQR) 8.7–28, mean 19/100PY). In the eleven studies only including current/recent PWID the incidence was higher than in the remaining studies (median 26/100PY IQR 9.4–35 vs. median 12/100PY IQR 9.0–16), and ranged from 5.2/100PY years to 42/100PY (both UK) [74], [85]. The two studies in custodial settings were small, with only eight (25/100PY, Denmark, ever PWID) [86] and 69 (12/100PY, UK, ever PWID) [76], [87] participants. The three small studies of HCV re-infection reported incidences of 6.9/100PY following a negative HCV RNA test result (N = 347, UK, ever PWID) [83], 3.4/100PY (N = 11, Netherlands, ever PWID) [88] and 0–4.1/100PY following treatment induced viral clearance (N = 18, Germany, ever PWID) [89].

### Chronicity

#### Study questions

What is the prevalence of chronic HCV infection among anti-HCV positive PWID in Europe? How does chronicity vary by setting, demographics, duration of injecting, and co-infection status?

#### Main findings

**Figure 3:**
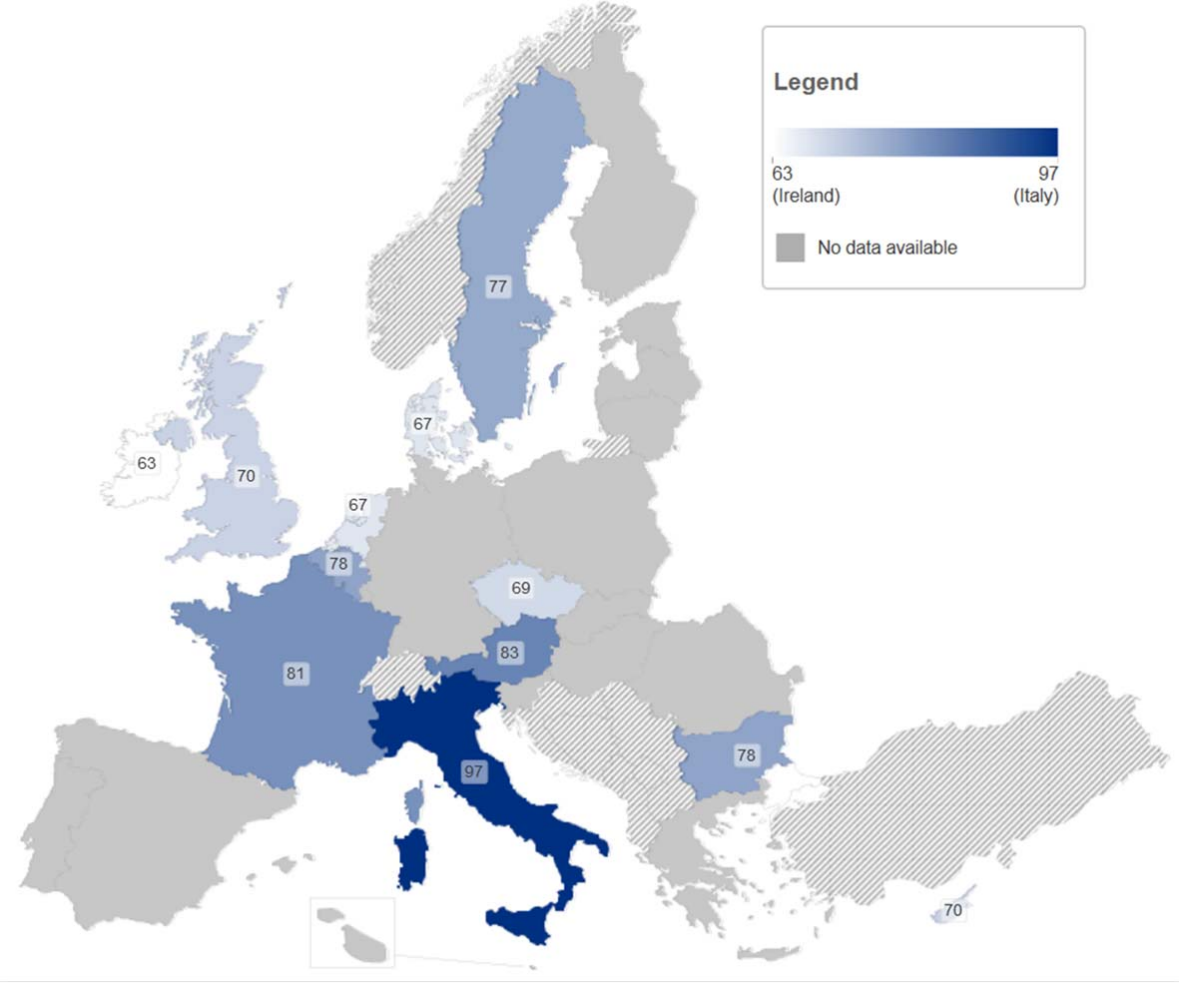
Chronicity of HCV: RNA prevalence (%) among antibody-positive PWID.

Available data on HCV-RNA rates in anti-HCV positive PWID show considerable variation (Table 4, Figure 3).

#### Studies included

Twenty-seven studies met the inclusion criteria [81], [82], [90]–[116] from fourteen countries (Table 4). These investigated the prevalence of HCV-RNA in 28 populations which included 10,263 anti-HCV positive PWID. Three additional studies were included that investigated the development of chronic infection in 98 PWID acutely infected with HCV [117]–[119]. (Table S4 in Web-appendix S2).

#### Study design

Nine cohort studies [100], [103], [105], [109], [113], [115], [117]–[119], and 21 cross-sectional studies tested HCV-RNA using PCR as a marker for chronic infection [81], [82], [90]–[99], [101], [102], [104], [106]–[108], [111], [112], [114], [116].

#### Population

PWID were recruited in drug treatment centres, general practices, gastroenterology and hepatology units, infectious diseases and genitourinary medicine clinics, and in the community.

#### Findings

The level of chronic infection in anti-HCV positive PWID ranged between 53% and 97% with a median of 72% (IQR 64–81%). The proportion of acute HCV infections among PWID progressing to chronic infection varied between 0% and 56%.

Based on seven studies with mean age [90], [91], [96], [103], [106], [111], [115] and four studies with mean duration of injecting drug use of the anti-HCV positive PWID [91], [96], [111], [113] a significant positive linear relation was observed between chronicity rate and both mean age of the population (Regression coefficient = 0.14; 95% CI: 0.00–0.28; P = 0.046) and mean duration of injecting drug use (Regression coefficient = 0.18; 95% CI: 0.02–0.34; P = 0.026). Based on the results of three studies [92], [110], [116] a significant relationship between HIV-co-infection (OR 1.67, 95% CI 1.07–2.60; P = 0.0025) and chronicity rate was observed. Based on the results of five studies a statistically significant association was found between male gender (OR 1.64, 95% CI 1.06–2.55; P = 0.016) [90], [91], [108], [110], [116] and chronicity rate. Only two studies had examined variation in the prevalence of HCV chronicity by HBV serostatus, and found no association (OR 0.99, 95% CI 0.63–1.57; P = 0.978) [91], [110]. Furthermore, the pooled average chronicity prevalence in the studies conducted in gastroenterology or hepatology units (84%, 95% CI: 74–91) was significantly higher than in the studies conducted in other settings (71%, 95% CI: 67–75) (Q-value = 5.292; df = 1; P< 0.021). Finally, no geographic trends could be detected.

### Genotypes

#### Study questions

What is the genotype distribution in PWID in Europe, and is it changing over time? What is the proportion of traditionally ‘difficult to treat’ genotypes (1 and 4)?

#### Main findings

HCV genotypes 1 and 3, (subtypes 1a and 3a), are the most commonly identified among PWID in Europe. Genotype 4 may be increasing. The proportion of the more ‘difficult to treat’ genotypes (1+4) showed large variation (17–91%, median 53%, IQR 43–62%).

#### Studies included

**Figure 4:**
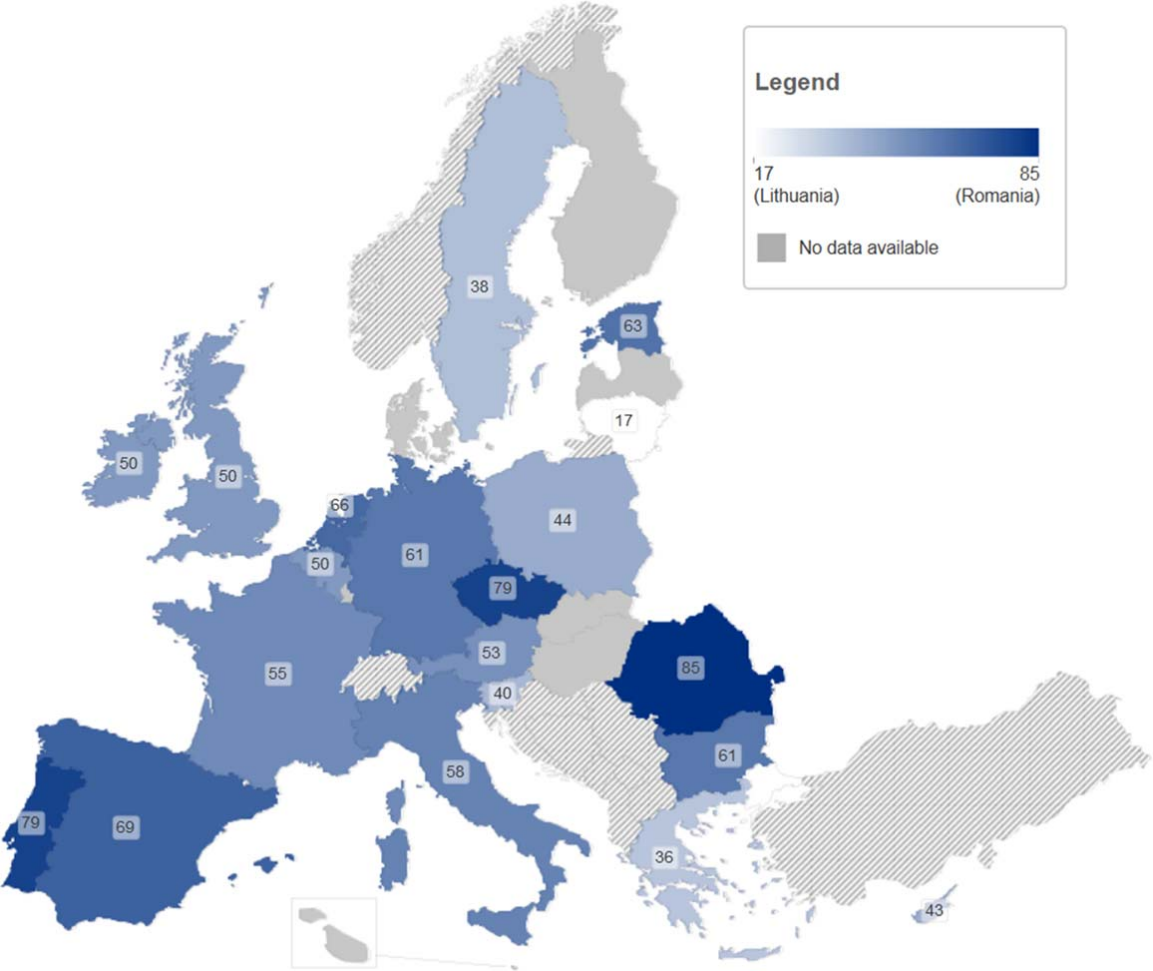
Proportion (%) of HCV infections among PWID that are genotypes 1 or 4.

43 studies met the inclusion criteria. Data were available from 20 European countries [81], [82], [91], [92], [94]–[97], [102], [103], [106], [108], [111]–[113], [115], [120]–[147]. (Table 4, Figure 4 and Table S5 in Web-appendix S2).

#### Study design

Eighteen cohort and twenty-five cross-sectional studies. HCV infection was mainly confirmed by enzyme immunoassays, immunoblot assays and RT-PCR. HCV genotypes/subtypes were determined by reverse hybridization assay and restriction fragment length polymorphisms (RFLPs) assay, sequencing and other PCR-based methods.

#### Population

A total of 6,488 HCV-infected PWID were genotyped or subtyped (range of subjects: 11–865). PWID were: a) enrolled in drug treatment, screening or national survey programs, b) regularly monitored in drug treatment centres, or c) hospitalised in or referred to specified units.

#### Findings

HCV subtype 1a dominates in Portugal [140], Spain [142], [143] and The Netherlands [113], [144], while it is also common in the Czech Republic [97] and the UK [81], [115], [145]. HCV subtype 1b prevails in Bulgaria [95], Czech Republic [122], Estonia [123] and Romania [111].

HCV genotype 2 is relatively uncommon with the exception of Greece [106], [132], Lithuania [138], Sweden [112] and the UK [82], [145], [147]. Genotype 3 predominates in Greece [106], [129], [130], [133], [134], Poland [139] and Slovenia [141] and is common in Austria [120], Belgium [91], France [103], [125] and Italy [136], [137]. Subtype 3a dominates in Belgium [92], Cyprus [96] and France [102], [124]. In Ireland, HCV genotypes 1 and 3 are equally distributed (48%) [108].

Levels of genotype 4 are particularly high in Southern European countries (Greece [130], [133], [134], Italy [136], [137], Portugal [140] and Spain [142], [143]) compared to Western ones (France [102], [125], Belgium [91], [92] and The Netherlands [113], [144]. The lowest rates of genotype 4 were reported in Lithuania [138], Sweden [112], Czech Republic [97], [122], and UK [146], [147]). Genetic diversity of genotype 4 suggests that this genotype is emerging among PWID and among the general population (e.g. 4a in Portugal [140] and Germany [127], [128], 4d in Portugal [140] and The Netherlands [113] and 4f in Italy [137]). Overall, the proportion of ‘difficult to treat’ genotypes (1+4) varies strongly, from 17% in Lithuania to 76–91% in the Czech Republic (Table 4) with a median of 53% (range 17–91%, IQR 43–62%).

Increasing levels of mixed infections are observed, by either different HCV genotypes (Italy 1b/3a [137], Germany 2a/3b [128] and Sweden 1a/2b, 1b/2a, 1a/4, 1b/2b [112]) or different subtypes of the same genotype (Spain 2a/2c and 4c/4d [143], Belgium 4c/4d [92], The Netherlands 2a/2b [113], and Sweden 2a/2b [112]). This is most likely due to the implementation of newer line probe assays with higher capability in detecting HCV subtypes.

### HIV co-infection

#### Study questions

What is the prevalence of HIV among PWID with HCV infection (HIV-HCV co-infection prevalence) in Europe?

#### Main findings

**Figure 5:**
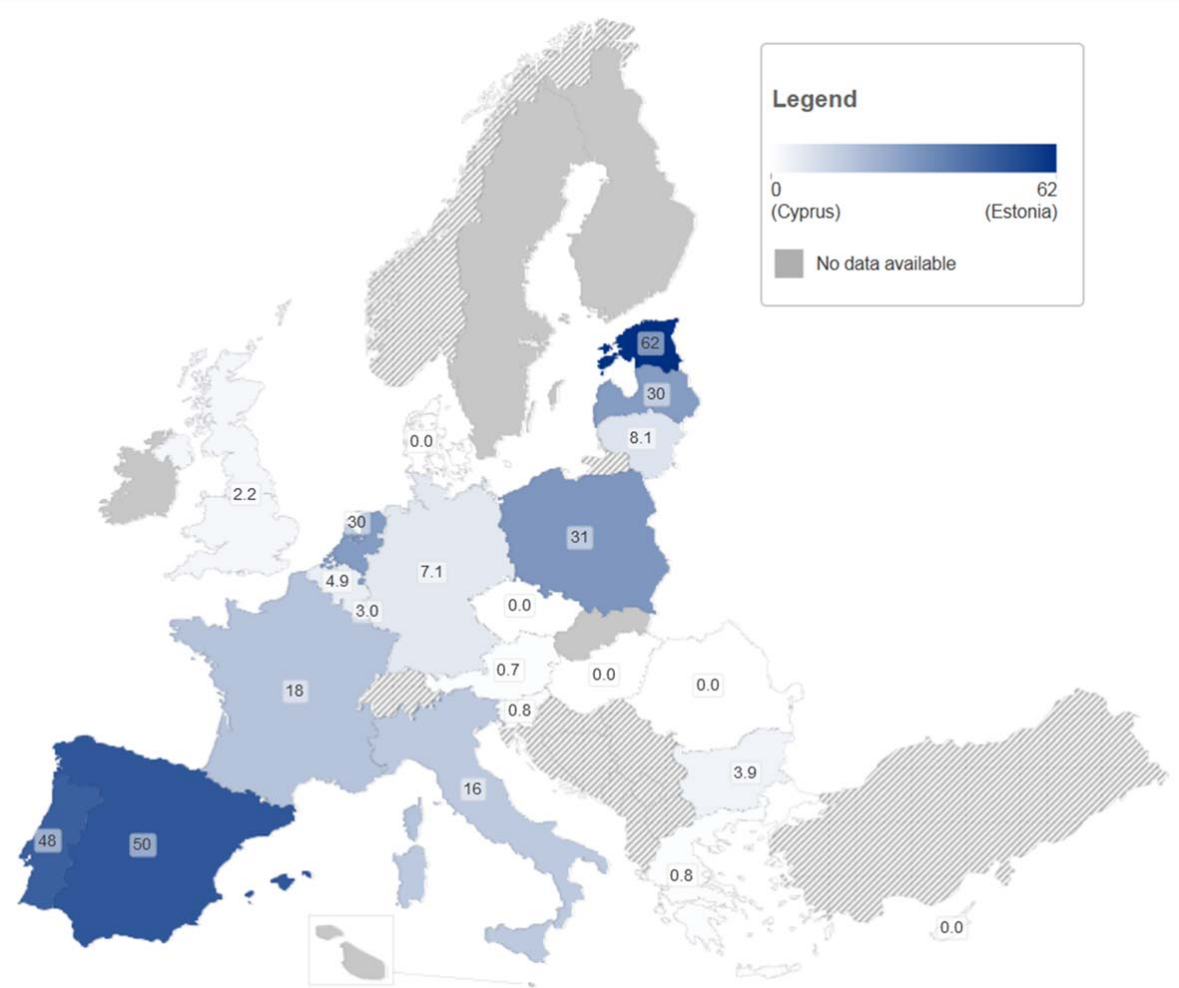
Proportion (%) of HCV-infected PWID that are co-infected with HIV.

Available data for 22 countries in Europe suggest considerable variation in the HIV-HCV co-infection prevalence (0–70%, median 3.9, IQR 0.2–28) among PWID (Table 4, Figure 5), with this being correlated (correlation coefficient = 0.98) with the HIV prevalence among PWID, but generally a median of 15% (IQR 0.0–49%) relatively greater.

#### Studies included

Sixty-two studies [22], [61], [71], [82], [86], [92], [96], [98], [99], [103], [106], [109], [111], [116], [118], [122], [129], [132], [134], [146], [148]–[189] met the inclusion criteria giving 80 HIV-HCV co-infection estimates. (Table S6 in Web-appendix S2).

#### Study design

Studies either involved diagnostic testing or cross-sectional samples of PWID from a variety of settings involving different sampling methods such as respondent driven sampling, exhaustive sampling and snowball sampling. HCV infection status was not confirmed by RNA status in many studies and so antibody prevalence was used across all studies.

#### Population

PWID were recruited from drug treatment centres, opiate substitution treatment centres, needle and syringe programmes, hospitals, and prisons.

#### Findings

Many European countries had multiple estimates of HIV and HCV prevalence but few recorded HIV-HCV co-infection prevalence (HIV prevalence among HCV antibody positives). Estimates of HIV-HCV co-infection prevalence were available for 22 countries in Europe with thirteen countries having multiple estimates (Belgium, Czech Republic, Denmark, France, Germany, Greece, Hungary, Italy, Latvia, Poland, Slovenia, Spain and UK). The HIV-HCV co-infection prevalence ranged between 0% and 70% in the different countries (median 3.9, IQR 0.2–28). Co-infection prevalences were low (< = 4%) in 11 countries (Austria, Bulgaria, Cyprus, Czech Republic, Denmark, Greece, Hungary, Luxembourg, Romania, Slovenia and UK), moderate (4 to 15%) in three countries (Belgium, Germany and Lithuania) and high (>15%) in eight countries (Estonia, France, Latvia, Italy, Netherlands, Poland, Portugal and Spain). As expected the HIV-HCV co-infection prevalence is higher in settings with higher HIV prevalence, with a strong linear correlation existing between each survey’s HIV prevalence estimate and the corresponding HIV-HCV co-infection prevalence estimate (correlation coefficient = 0.98), with the HIV-HCV co-infection prevalence being a median of 15%(IQR 0.0–49%) relatively greater than the HIV prevalence. No clear relationship existed between HCV prevalence and the HIV-HCV co-infection prevalence.

### Diagnosis

#### Study question

What percentage of HCV-infected PWID are undiagnosed in Europe?

#### Main findings

**Figure 6:**
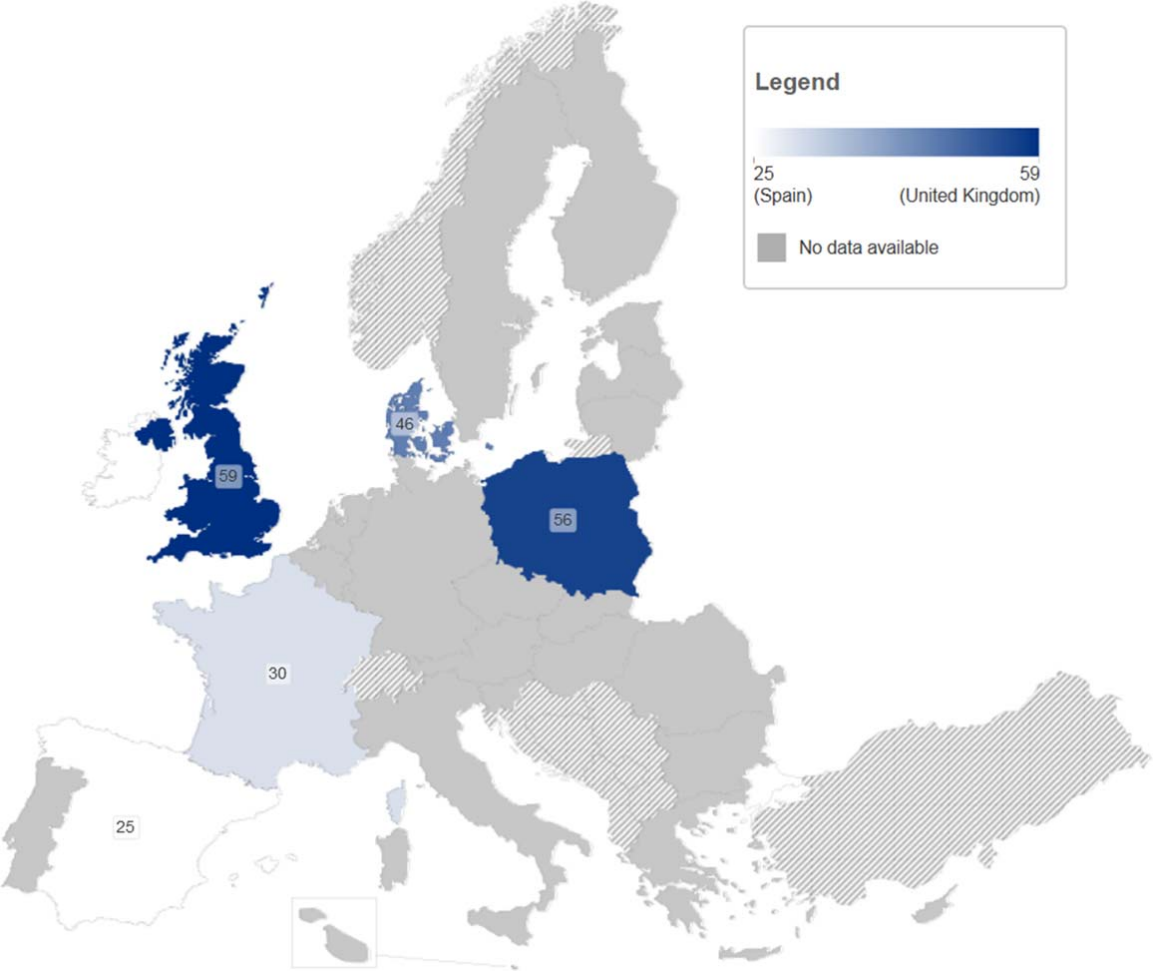
Proportion (%) of HCV positive PWID (antibody or RNA) undiagnosed.

Among the studies included, a high proportion of HCV infections in PWID were undiagnosed (median 49%, range 24–76, IQR 38–64) (Table 4, Figure 6).

#### Studies included

Eleven studies reported on the proportion of PWID with undiagnosed hepatitis C infection from five countries in the EU [114], [170]–[172], [180], [190]–[195]. (Table 4 and Table S7 in Web-appendix S2).

#### Study design

Ten cross-sectional studies and one retrospective cohort study. All studies were observational and performed in non-clinical settings.

#### Population

Ten studies among PWID in specialised treatment centres and other drug services, one study among PWID attending general practices.

#### Findings

The proportion of infections in PWID that were previously undiagnosed ranged from 24% to 76% with a median of 49% (IQR 38–64%, n = 13,561).

### Treatment

#### Study question

What is the proportion entering antiviral treatment among diagnosed cases of chronic HCV infection in PWID?

#### Main findings

**Figure 7:**
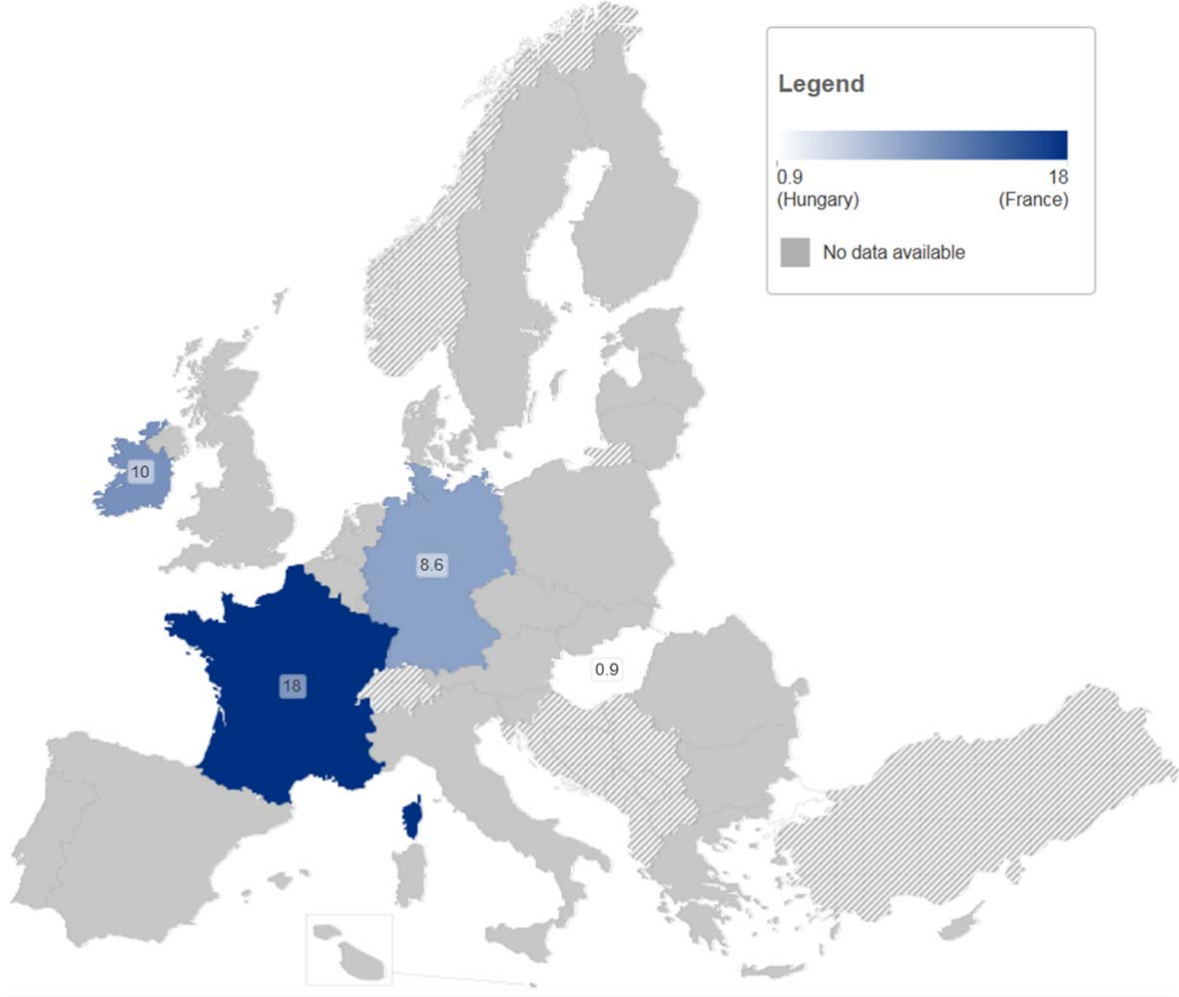
Proportion (%) of HCV-infected PWID entering antiviral treatment in observational studies in non-clinical settings.

In six observational studies with non-clinical recruitment settings, the proportion of PWID diagnosed with chronic HCV infection that started antiviral treatment was generally low, at 1–19% (median 9.5, IQR 3.5–15) (Table 4, Figure 7).

#### Studies included

Twenty-six studies from 11 countries fulfilled the inclusion criteria [22], [59], [79], [103], [105], [107], [114], [115], [126], [131], [146], [152], [165], [188], [196]–[206] (Table 4 and Table S8 in Web-appendix S2).

#### Study design

Eight were retrospective cohort studies; 11 were prospective cohort studies; one a randomised controlled trial; one a semi-experimental intervention study and five were cross-sectional studies. Overall, 16 were observational, whereas 10 were intervention studies (one study included both observational and intervention data).

#### Population

PWID were either patients attending hospitals or specialist services for hepatitis treatment (‘clinical’ – eight studies), or recruited through PWID specific services, general practice and/or community settings (‘non-clinical’ – 18 studies).

#### Findings

The proportion of PWID with diagnosed chronic infection entering antiviral treatment was 1–19% (median 9.5, IQR 3.5–15) in six non-clinical observational studies (four countries, total sample size 3,017) [105], [107], [152], [196], [199], [201]. An increasing proportion is seen by setting and study type with progressively selected study populations, with a median of 23% (IQR 17–31) in nine intervention non-clinical studies, 28% (IQR 24–42) in seven observational clinical studies [22], [103], [115], [131], [198], [200], [204] and 47% in one intervention clinical study [126]. Four studies with non-clinical recruitment settings provided a proportion of antibody positive PWID self-reporting having ever been treated, with a median of 4.7% (3.4–37, IQR 4.3–13, n = 868) [79], [146], [165], [202]. Three observational studies with non-clinical recruitment settings provided the proportion of diagnosed PWID referred to a specialist for treatment evaluation (median 57%, range 9.0–59) [79], , as did four intervention studies in non-clinical settings (median 59%, range 21–93, IQR 40–78) [146], [197], [205], [206].

### Burden of disease

#### Study questions

What estimates exist of the future burden of disease among PWID with HCV in Europe?

#### Main findings

The crude mortality rate (CMR) for all-cause mortality ranged from 2.1–12 cases /100PY. Modelling studies project an increase in the burden of liver disease. HCV treatment could reduce this burden and is cost-effective (Table 4).

#### Studies included

Four observational studies, two modelling studies and one cost-effectiveness study [177], [207]–[211] (Table 4 and Tables S9–S10 in Web-appendix S2).

#### Study design

All cohort studies had a prospective design and one also included retrospective data. Study settings varied from single centre to nationwide.

#### Population

PWID were recruited from HIV centres and through drug treatment centres.

#### Findings

During 33,284 PY of follow-up 895 of 5,340 PWID died. The prevalence of HIV co-infection varied between 16% [209] and 100% [207], [208].

All studies reported all-cause CMR, ranging from 2.1 to 12 cases /100PY, (Table S9 in Web-appendix S2). A Danish study [207] found comparable CMR for those with chronic HCV and spontaneous resolvers, whereas a study from The Netherlands [209] observed a two-fold higher CMR for chronically HCV-infected PWID compared to spontaneous resolvers. The Danish study [207] reported a >4 times higher CMR than the other studies. Two studies reported liver-related CMR [207], [209], of 0.11 and 3.0/100PY respectively.

Two modelling studies were included, from The Netherlands [210] and the UK [211], (Table S10 in Web-appendix S2). Between 2011 and 2025, the HCV-related liver disease prevalence in The Netherlands was projected to rise by 36% [210]. In Scotland, [211] a 56% increase in cirrhosis and 64% in moderate liver disease between 2010 and 2025 was projected. Both studies showed that HCV treatment could reduce the future liver disease burden.

One cost-effectiveness analysis from the UK, based on a dynamic model of HCV transmission and disease progression [28], found that in a steady state epidemic with HCV prevalence among PWID of 20% or 40%, HCV treatment of PWIDs was more cost-effective than treating non/ex-PWIDs with an incremental cost-effectiveness ratio of 599 and 2,920 euro, respectively, per quality adjusted life year as compared to no treatment.

## Discussion

Our study suggests that availability of key data for informing the scale-up of HCV treatment for PWID in Europe is highly variable, but diagnosis and treatment uptake remain low. To our knowledge, this is the first attempt to provide a comprehensive and comparative review of data for HCV treatment scale-up among PWID in a large number of countries.

While most countries have information on the genotype distribution and HIV-HCV co-infection prevalence in PWID (22/27 countries), only six countries have estimates for five or more of the seven topic areas reviewed here (Denmark, France, Ireland, The Netherlands, Spain and UK). In addition, available data are often based on selected subpopulations (e.g. clinical) and local studies, which might not be representative for all PWID living in a country, while in some of the topic areas recent data (collected since 2006) are scarce. Our review reveals serious gaps in data availability and comparability, suggesting that many countries in Europe may not yet have invested sufficiently in studies or surveillance systems to guide HCV treatment and prevention policies for PWID. Moreover, although we did not systematically evaluate the quality of data, we observed large differences in methods and definitions, likely affecting comparability across data sources [212]. Importantly, the limited data suggest that overall there are poor levels of diagnosis and treatment uptake among HCV-infected PWID.

Although diagnosis is a pre-condition for treatment entry and under-diagnosis an important reason for non-treatment [34], we found information on the undiagnosed fraction in PWID in only five countries (19%). The proportion undiagnosed was over 50% in five of the 11 studies (overall median 49%, range 24–76%), while methods were not always clear or comparable.

More countries (11/27, 41%) had data on antiviral treatment access among PWID (note: among those diagnosed). However, only four countries had studies in non-clinical settings that were non-interventional (i.e. did not specifically attempt to enhance treatment access). Among the studies in these countries, the median proportion of diagnosed PWID who had actually started treatment was 9.5% (range 1–19%). Interestingly, the proportion entering treatment increased with the level of selection of the study population (with a median of 23% in nine non-clinical intervention studies, 28% in seven clinical observational studies and 47% in one clinical intervention study) suggesting that potentially higher treatment rates may be achieved with specific interventions. However, even in the most selected intervention studies a large proportion of diagnosed PWID remained untreated.

The median proportion of antibody-positive PWID who self-reported lifetime treatment uptake for HCV in four non-clinical studies (4.7%), although within a large range (3.4–37%), was roughly similar to that in two studies in the US, which found a lifetime uptake of 4.8% among RNA-positive PWID [20] and of 6% among antibody positive PWID [15]. An Australian study found a self-reported life-time treatment uptake of 10% and an increasing trend in the annual treatment uptake (0.5% in 1999–2% in 2011) among HCV antibody-positive PWID [19]. A recent study in UK used a novel method based on laboratory data to estimate HCV treatment uptake and outcomes, however was unable to obtain information by risk group [213].

Referral of chronically infected PWID from non-specialist to specialist care was found to be incomplete, explaining part of the low treatment access. However, this was reported by only three of the observational studies in non-clinical settings (median 57%, range 9–59) and was found to be similar in four (non-clinical) intervention studies (59%, range 21–93). The limited treatment uptake may finally also be due to the absence of national treatment policies for PWID (Table 4) and/or a decision to wait for the new potent direct-acting antiviral agents (DAA).

Treatment outcomes are determined by patient characteristics, which traditionally include HIV co-infection and HCV genotype [22], [36], [214]. Although in principle both may soon be less relevant [36], [214], [215], it remains to be seen if the much higher costs of treatment for the unfavourable genotypes and DAA may hamper their scale-up for PWID in many countries [30], [216]. Data on these two topic areas were more abundant with 20 countries (74%) having information on genotype distributions and 22 on HIV co-infection in PWID. The data suggest important variation in genotypes across countries in Europe, with large variation in the proportion of ‘difficult to treat’ genotypes among PWID (sum of percentages of genotypes 1 and 4), ranging from 17% in Lithuania to 76–91% in the Czech Republic (median 53%). Genotypes 1 and 3, especially subtypes 1a and 3a, are common and have demonstrated exponential growth during the 20^th^ century [217]. Genotype 4 has been spreading in Western Europe since the 1960s [217]. It appears to be genetically diversifying and increasing in particular among PWID in Europe [46], [113], [128], [137], [140], [144], [218]–[220]. Countries with predominance of genotypes 1 and high or increasing levels of genotype 4 face more difficulties in treating PWID with peginterferon and ribavirin. High levels of HIV co-infection (over 4%) were found in 11 countries, with levels over 15% being found in eight of these (Estonia, France, Italy, Latvia, The Netherlands, Poland and Portugal, Spain).

Future treatment needs among PWID, especially for ‘treatment as prevention’, will not only depend on the current prevalence of chronic infection, but importantly on the incidence of new infections and re-infection. Incidence estimates of new infections in PWID based on direct methods were located for only nine countries (33%) and suggested wide variation in infection rates (2.7–66/100PY overall and 5.2–42/100PY among current and recent PWID) whereas just three countries have reported re-infection rates (0–6.9/100PY), although these are likely to be underestimates of the real re-infection rate [53], [221]. The countries at highest (Sweden) and lowest (Netherlands) incidence appear to confirm a previous, prevalence-based, analysis suggesting a link with prevention policies (Figure 2) [42]. Chronicity levels among PWID were found for 12 countries (44%) showing a large range (53–97%). Multiple studies at these extremes (six below 60%, two above 90%, out of 28) suggest substantial differences in progression to chronic infection may exist between countries, which might be explained by differences in gender and HIV co-infection prevalence distributions between study populations and the setting of the study, and may impact future treatment needs.

To actually estimate future treatment needs, modelling studies are important, particularly for assessing the potential impact of treatment on prevention. Only two of the 27 countries appear to have carried out a modelling study to estimate the effect of HCV treatment on the future burden of disease. Without treatment, a study in The Netherlands (Amsterdam) projected a 36% increase in the burden of liver disease between 2011 and 2025, whereas in Glasgow this was projected to be 56–64% for 2010–2025. Both studies included competing mortality in their model and showed that HCV treatment would substantially reduce this liver disease burden.

Mortality in PWID with HCV infection is dependent on competing mortality (e.g. HIV or drug-related death [222]–[224]) and duration of persistent HCV infection. The all-cause mortality rates were estimated at 2.1–2.4/100PY in Spain and The Netherlands, but were 12.2/100PY among HIV co-infected PWID in Denmark. The high rate in the Danish study could be explained by high rates of overdose mortality [49] or differences in cART initiation, given that a Spanish study reported a CMR of 2.4/100PY among HIV co-infected PWID during a comparable study period. This suggests significant country differences for PWID with HCV, in line with findings on mortality among all PWID [224] and the importance of obtaining country-specific mortality estimates. Available data on the morbidity and mortality risk due to HCV among PWID are scarce but are urgently needed for future planning.

The data reported here are related to the EU and therefore our results are most probably not generalisable to other regions globally. However, it is likely that data availability in low and middle income countries will be lower, despite the often more serious epidemiological situation among PWID regarding blood-borne infections e.g. in Eastern Europe and Central Asia [33], [43], [225]–[227]. Our study provides a framework and methodology for combining a series of complementary systematic reviews using standardised and validated methods that may be applied to other regions, or topic areas, where a comprehensive view may be beneficial [57], for example when the aim is to support national public health policies. We are unaware of a similar systematic review of the literature comparing multiple related topic areas around a key public health issue in a large number of countries.

Our study is subject to important limitations. We have primarily focused on published studies, available through the MEDLINE, EMBASE and the Cochrane Library databases, which are subject to publication bias and delay. To minimise this problem, we contacted a network of drug and infectious disease experts in Europe, to identify missed studies and we included publications in all languages. Another limitation relates to our inclusion criterion of having an unselected population of PWID with regard to gender or HBV/HIV infection and to restricting our review to data of PWID (ever injectors) only. There are many more reports on populations of people who use drugs where injection status is not known or that are limited to HIV co-infected patients which we could mostly not include, although we believe by being restrictive we may have minimised potential bias. We were also mostly (except for Incidence) unable to distinguish studies based on ever or recent (active/current) injectors as this information was usually not available. However, as most of our outcomes relate to HCV chronicity we believe the data here presented are not seriously affected by recent injecting status of the ever PWID in our study and ever-PWID are the more appropriate group to study. Data in the Diagnosis and Treatment sections would ideally have been adjusted for duration of follow-up in the studies, however these data were mostly not available for the data we extracted. Our data are further limited regarding the comparability of studies found. In particular the geographic coverage of studies was usually partial, with few national studies (Table 4), and they were often undertaken in health services; so participants may not be representative of all PWID. Thus some of the differences between and within countries found here may reflect differences in study design and methodologies rather than true differences. Finally, we selected the topic areas for our review on the basis of informed expert discussions among the authors on what are the key data elements for treatment scale-up that are not available from routine monitoring (Table 1 and Table S2). Further work may be necessary to develop consensus guidance on the information needed to guide HCV treatment policies.

**Figure 8:**
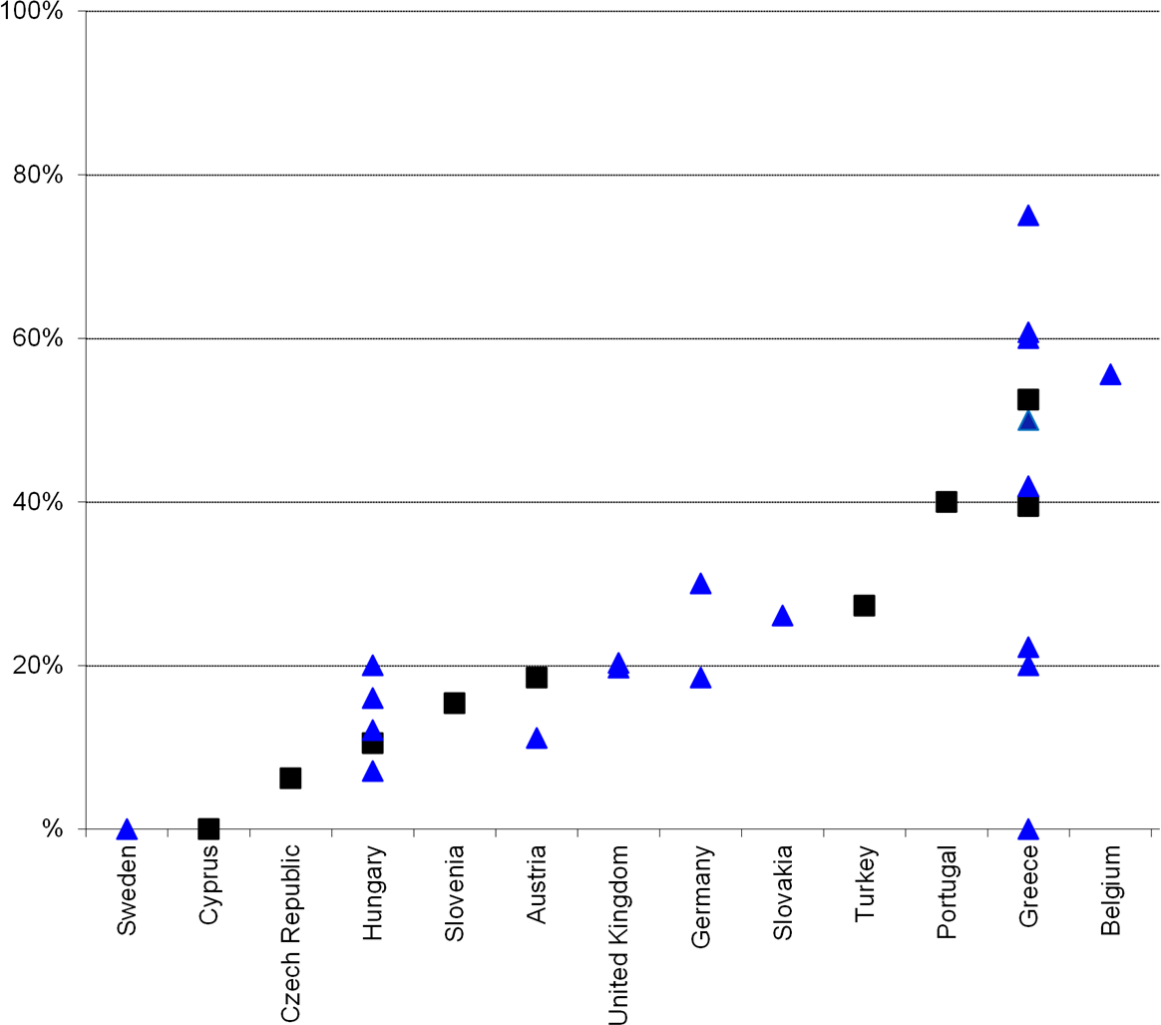
HCV antibody prevalence (%) among PWID injecting<2 years in the EU, 2006–2011. Note Figure 8: Source EMCDDA, 2013. (http://www.emcdda.europa.eu/stats13#inf:displayTables); black squares are data with national coverage, blue triangles are data with sub-national (local, regional) coverage.

**Figure 9:**
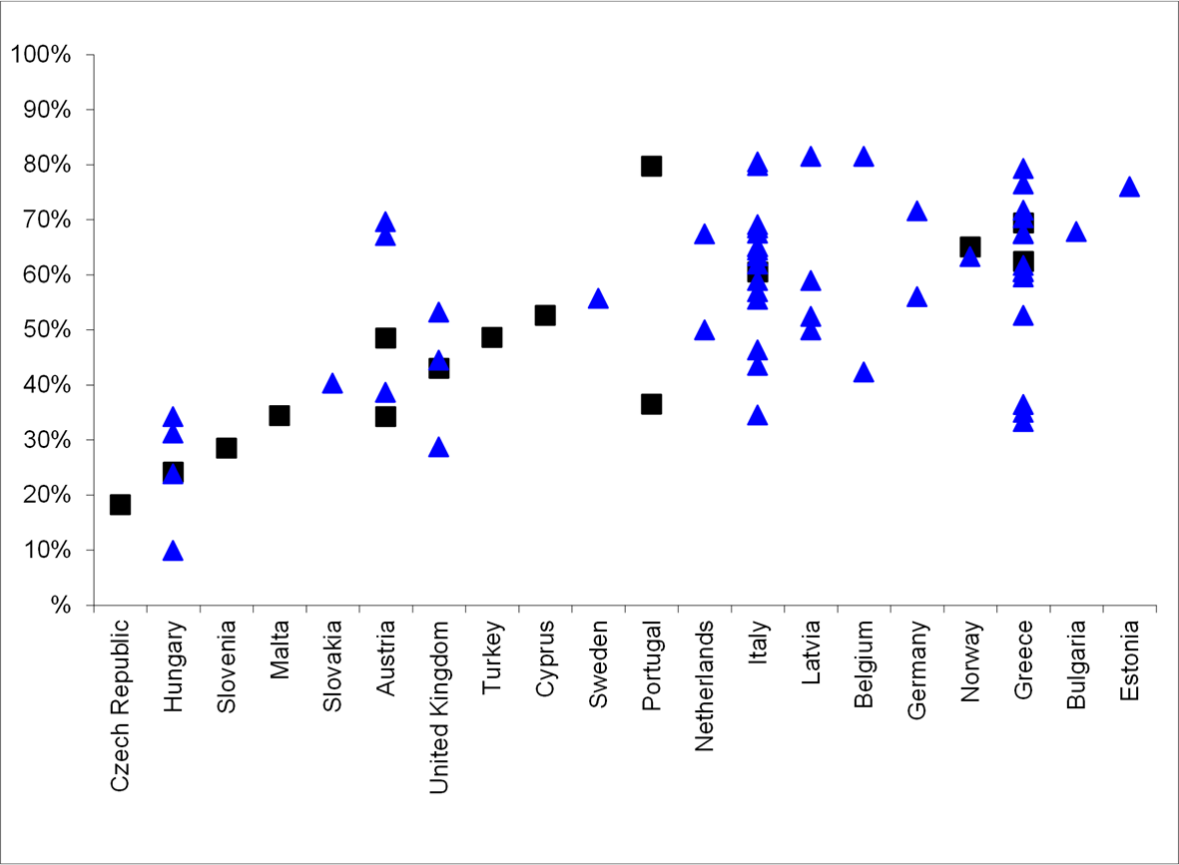
HCV antibody prevalence (%) among PWID in the EU, 2006–2011. Note Figure 9: Source EMCDDA, 2013 (http://www.emcdda.europa.eu/stats13#inf:displayTables); black squares are data with national coverage, blue triangles are data with sub-national (local, regional) coverage.

In this review we focused on data from the literature, as a complement to data on HCV infection that is routinely collected by EU bodies, and is likely to be used in national policy decision making. The routine data include hepatitis C notifications, HCV antibody prevalence among PWID, PWID population size, HIV prevalence in PWID (as a proxy for co-infection levels), and the provision and coverage of needle and syringe programmes and opioid substitution treatment as primary HCV prevention measures for PWID (Table S2 in Web-appendix S1). Although some of these data are widely available, for example most countries have an estimate of HCV antibody prevalence among PWID [43], [44], [49], other available data can be difficult to interpret. Acute hepatitis notifications are likely to represent only a very small proportion of real incidence, due to the asymptomatic nature of acute HCV infection and underreporting [228], while trends in chronic infections cannot be easily interpreted due to the long time to diagnosis and their dependence on testing patterns, in addition to potentially serious underreporting [4], [XPATH ERROR: unknown variable "rids-text".] and incomplete availability of risk information. Of particular interest is the HCV antibody prevalence in new injectors (injecting for less than 2 years) (Figure 8). This may provide a relatively cost-effective indicator of levels of new infection in PWID [44], [192], as an addition to the regular prevalence data (Figure 9), particularly if it is supported and regularly validated by incidence studies using direct methods as here reviewed, or, in addition, indirect methods [211], [230]. Finally, it should be noted that, although population size estimates of PWID are routinely monitored in Europe [49], few countries have estimates that are reasonably recent. This highlights an important need for improvement, given that population size estimates enable converting data from different sources into the absolute numbers needed for planning.

In conclusion, the availability of key data for informing the scale-up of HCV treatment among PWID in individual European countries is highly variable. Our study suggests that large proportions of HCV-infected PWID remain undiagnosed, and of those diagnosed, only one in ten receive antiviral treatment. Stronger national and international efforts, including operational research and collection of key data on PWID with HCV, are needed to develop sound HCV treatment policies for PWID in Europe.

## Supporting Information

Web-appendix S1 **Study protocol and Table S2 – availability of routine data.** References cited: [231], [232].
(DOCX)

Web-appendix S2 **Tables S3 to S10 – full data per topic area.**
(XLSX)

Web-appendix S3 **Additional detail on the Chronicity analyses.**
(DOCX)

Web-appendix S4 **PRISMA checklist.**
(DOCX)
