# Supplementary material for: Hepatitis C Virus Infection Epidemiology among People Who Inject Drugs in Europe: A Systematic Review of Data for Scaling Up Treatment and Prevention
Source: PLoS One. 2014 Jul 28;9(7):e103345. doi: 10.1371/journal.pone.0103345 (PMC4113410; doi:10.1371/journal.pone.0103345)
Supplement: Web-appendix S1 — Study protocol and Table S2 – availability of routine data. References cited: [231], [232]. (DOCX) [file pone.0103345.s001.docx]

Web-appendix S1: Study protocol and Table S2

Study Protocol

I. SEARCH STRINGS USED

General format of searches

Search string

((general search string) AND (topic search string) NOT (countries excluded))

Limits

Between 1/1/2000 and 31/12/2012, no language or other restrictions were used

Geographic coverage

EU 27: Austria, Belgium, Bulgaria, Cyprus, Czech Republic, Denmark, Estonia, Finland, France, Germany, Greece, Hungary, Ireland, Italy, Latvia, Lithuania, Luxembourg, Malta, Netherlands, Poland, Portugal, Romania, Slovakia, Slovenia, Spain, Sweden, United Kingdom

a) PUBMED

General search string

(“Substance Abuse, Intravenous”[Mesh] OR “IDU” OR “IDUs” OR “PWID” OR “IVDU” OR “IVDUs” OR “intravenous drug” OR “injecting drug” OR “intravenous substance” OR “Injection drug” OR “inject drugs”) AND (“Hepatitis C”[Mesh] OR “hepatitis C” OR “HCV”)

Topic search strings

1. "incidence"
2. "genotype"[tiab] OR “subtype"[tiab] OR “molecular epidemiology” [tiab]
3. HIV or "HIV"[Mesh] or ”hiv*”
4. HCV-RNA[All Fields]^[[1]](#footnote-1)^ OR ("genotype"[MeSH Terms] OR "genotype"[All Fields]) OR persistence[All Fields] OR ("viraemia"[All Fields] OR "viremia"[MeSH Terms] OR "viremia"[All Fields])
5. “test"[tiab] OR "prevalence"[tiab] OR "proportion"[tiab] OR "referral"[tiab] OR "trend"[tiab] OR "screening"[tiab] OR "diagnostics"[tiab] OR "surveillance"[tiab] OR "unidentified"[tiab] OR "diagnosis"[tiab] OR "undiagnosed"[tiab]
6. "antiviral"[tiab] OR "treatment"[tiab] OR "therapeutics"[tiab] OR "access to treatment"[tiab]
7. Cost-effectiv* [tiab] OR burden [tiab] OR daly [tiab] OR qaly[tiab] OR morbidity [tiab] OR mortality[tiab] OR "Cost of Illness"[Mesh] OR illness cost*[tiab] OR incremental cost-effectiveness ratio [tiab] OR Cost-Benefit Analysis [Mesh]

Countries excluded automatically (all countries in the Mesh terms list not included in the study were listed, other countries were excluded manually)

NOT (haiti* OR brazil* OR iran* OR irak OR iraq* OR syria* OR malawi* OR india* OR nepal* OR japan* OR "South Africa"[MeSH Terms] OR "Developing Countries"[Mesh] OR developing countr*[tiab] OR africa* OR singapore OR australia* OR arabia*[tiab] OR aboriginal*[tiab] OR china[tiab] OR hong kong[tiab] OR Thailand OR "Africa"[Mesh] OR "Americas"[Mesh] OR "Antarctic Regions"[Mesh] OR "Arctic Regions"[Mesh] OR "Asia"[Mesh] OR "Atlantic Islands"[Mesh] OR "Australia"[Mesh] OR "Baltimore"[Mesh] OR "Boston"[Mesh] OR "Chicago"[Mesh] OR "District of Columbia"[Mesh] OR "Los Angeles"[Mesh] OR "Moscow"[Mesh] OR "New Orleans"[Mesh] OR "New York City"[Mesh] OR “Philadelphia"[Mesh] OR "San Francisco"[Mesh] OR "Tokyo"[Mesh] OR "Arabia"[Mesh] OR "Armenia"[Mesh] OR "Byzantium"[Mesh] OR "Egypt"[Mesh] OR "Persia"[Mesh] OR "Commonwealth of Independent States"[Mesh] OR "Confederate States of America"[Mesh] OR "Korea"[Mesh] OR "Middle East"[Mesh] OR "New Guinea"[Mesh] OR "Ottoman Empire"[Mesh] OR "Russia (Pre-1917)"[Mesh] OR "Armenia"[Mesh] OR "Azerbaijan"[Mesh] OR "Georgia (Republic)"[Mesh] OR "Kazakhstan"[Mesh] OR "Kyrgyzstan"[Mesh] OR "Moldova"[Mesh] OR "Republic of Belarus"[Mesh] OR "Russia"[Mesh] OR "Tajikistan"[Mesh] OR "Turkmenistan"[Mesh] OR "Ukraine"[Mesh] OR "Uzbekistan"[Mesh] OR "Bosnia-Herzegovina"[Mesh] OR "Croatia"[Mesh] OR "Macedonia (Republic)"[Mesh] OR "Indian Ocean Islands"[Mesh] OR "Oceania"[Mesh] OR "Oceans and Seas"[Mesh] OR "Pacific Islands"[Mesh] OR "Andorra"[Mesh] OR "Albania"[Mesh] OR "Montenegro"[Mesh] OR "Serbia"[Mesh] OR "Gibraltar"[Mesh] OR "Iceland"[Mesh] OR "Liechtenstein"[Mesh] OR "Sicily"[Mesh] OR "Monaco"[Mesh] OR "San Marino"[Mesh] OR "Switzerland"[Mesh] OR "Transcaucasia"[Mesh] OR "Vatican City"[Mesh])

b) EMBASE

General terms

(((substance abuse or idu or idus or pwid or ivdu or ivdus or intravenous drug or injecting drug or intravenous substance) and (hepatitis c))

Topic specific terms

1. Incidence: and incidence
2. Genotypes: and (genotype or phylogenetic analysis or metaanalysis or systematic review)
3. HIV coinfection: and (hiv)
4. Chronicity: and ('viremia’ :exp OR 'viremia':ab,ti 0R 'viraemia':ab,ti OR 'persistence':ab,ti OR 'genotype':ab,ti OR 'genotype'/exp OR 'hcv-rna':ab,ti)
5. Diagnosis: and (test or proportion or referral or trend or screening or diagnostics or surveillance or unidentified or diagnosis or undiagnosed)
6. Care and treatment: and (antiviral or treatment or therapeutics or access to treatment)
7. Burden of disease: and (Cost-effectiv* or burden or daly or qaly or morbidity or mortality or Cost of Illness or illness cost* or incremental cost-effectiveness ratio or Cost-Benefit Analysis)

Countries excluded automatically (all countries in the Mesh terms list not included in the study were listed, other countries were excluded manually)

not (Haiti or Brazil or Iran or Irak or Iraq or Syria or Malawi or India or Nepal or Japan or South Africa or Developing countries or developing country or Africa or Singapore or Australia or Arabia or Aboriginal or China or Hong Kong or Thailand or Africa or Western hemisphere or Antarctica or Arctic or Asia or Atlantic islands or Australia or United States or Russian federation or Japan or Saudi Arabia or Armenia or Middle east or Egypt or Ussr or Korea or Papua new guinea or Ottoman empire or Azerbaijan or Georgia Republic or Kazakhstan or Kyrgyzstan or Moldova or Belarus or Tajikistan or Turkmenistan or Ukraine or Uzbekistan or Bosnia or Herzegovina or Croatia or Macedonia republic or Indian ocean or Pacific islands or sea or Andorra or Albania or Montenegro republic or Serbia or Gibraltar or Iceland or Norway or Liechtenstein or Monaco or San Marino or Switzerland or Vatican City State)).af.

c) Cochrane Library

Substance Abuse, Intravenous ;IDU ;IDUs ; PWID ;IVDU ;IVDUs; intravenous drug ; injecting drug; intravenous substance (in isolation and together with the use of OR) ; AND Hepatitis C or hepatitis c . Accessed 26-11-2012 (resulting in 200 articles once cleaned for relevance N=42)

II. Exclusion criteria

1.       THE STUDY POPULATION

a.      The study is conducted in populations residing outside the countries covered by this review

b.      The outcome(s) of interest is not reported for PWID separately

d.      The study is conducted in a selected group of PWID in terms of gender, HBV infection or HIV-infection. Also studies in autopsied bodies of PWID are excluded.

2.       THE SAME STUDY IS PRESENTED IN ANOTHER PUBLICATION

Publications are excluded if they do not provide further information on the same study. In case two articles provide complementary information they are both included and the information is combined for the analysis. In case samples overlap but cannot easily be combined then the largest sample is included (for example where national and sub-national data are available from the same study (i.e. same sample) then the national data are used).

2.      EXISTENCE OF CONTRADICTORY, CONFUSING OR ERRONEOUS DATA

Contradictory data within one or between multiple publications from the same study are excluded.

3.      SIZE OF SAMPLE

Sample sizes under n=10 are excluded unless they provide the only data in their country for that topic area.

 4.    DATE OF PUBLICATION AND STUDY DATE

Studies published before 2000 will be excluded. Data published after 01/01/2000 on events from before 1990 will also be excluded

III. Search strategy

a) Identification

1. Go through the tutorial of Pubmed (<http://www.nlm.nih.gov/bsd/disted/pubmedtutorial/>) and EMBASE (<http://trainingdesk.elsevier.com/embase>) before starting the work
2. Use the agreed general keyword string, countries exclusion string and time limits and send resulting numbers to EMCDDA, all should get the same number of hits
3. Identify topic specific key words and add them to the agreed general keyword string (see above)
4. Develop one final topic-specific string that is as sensitive and specific as possible, documenting each step for your own records (if you use multiple strings combine them in the final string using the OR operator, and save them for your own records). Check sensitivity e.g. by making sure a few known key articles are always included by the different versions of the search string
5. Check reference lists of main existing (systematic) reviews to see that all relevant papers are included, if not adapt search string
6. Make sure you save your final search string and include it in your methods description. You will need to use it for populating the PRISMA flowchart, send your final string of topic keywords to EMCDDA for sharing with others via Dropbox
7. Do the final search using the final search string
8. Export the results to reference manager (RM), save one RM database for each of the two top boxes of the flow diagram before removing duplicates, send to EMCDDA. Make sure that each RM database file is clearly named with regard to the collaborator, which box it corresponds and date using the following format “YournameRefsBox1v151012. rmd”, “YournameRefsBox2v161012.rmd”.
9. Remove duplicates retrieved from different sources, save the consolidated version as an RM database corresponding with the third box of flow diagram using the same file naming format, send to EMCDDA

b) Screening

1. Show only titles in RM and go through them excluding any title obviously not relevant, in a first quick scan exclude all studies for non-EU countries.
2. In a second scan exclude again only looking at titles all articles that are clearly not relevant content-wise, look at abstract in case of doubt, if doubt remains then include (be inclusive). Keep your own records on what articles were excluded in each step, this is easiest by saving them to a separate database (do not send these to EMCDDA).
3. Read all remaining abstracts and exclude articles that clearly are not useful, keep articles in case of doubt.
4. Save two RM databases corresponding with boxes four and five of the flow diagram (“records screened” and “records excluded”) using the same filenaming format as before, send to EMCDDA. These databases refer to the database for box 3 (which has all raw hits from the search string from all sources but excluding duplicates). Thus boxes four and five contain the results of the records screened and excluded, respectively, since box 3.
5. Make a short checklist of main inclusion criteria for further assessment, based on the main data elements of your data table, send to EMCDDA for comparison with the others (these checklists need to be standardised between topics!)
6. Assess all papers comparing abstract with checklist, in case of doubt include, keep a list of papers excluded in this phase with the criteria based on which they were excluded (e.g. keep your PICO at hand while you screen the articles so that when you list the reference under “Intervention” you know that the reason was not meeting the inclusion criteria for “Intervention” and so on). Save two RM databases corresponding with boxes six and seven of the flow diagram (“…assessed for eligibility” and “records excluded”) as well as the list of excluded papers with a code identifying the reason for exclusion, send to EMCDDA
7. Ask the second person involved to independently carry out steps 10 to 15, compare results and take final decision on discrepancies together. Send the revised RM databases again to EMCDDA giving them the same names as in step13 (i.e. these databases replace the databases sent in step 13).

c) Eligibility and Included

1. Retrieve all papers
2. Make fully detailed eligibility checklist with inclusion criteria based on your data table
3. Compare full papers with eligibility checklist / data table, keep a table of the papers excluded in this phase with exclusion criteria applied (papers as rows, criteria as columns and putting a ‘X’ or a short note in the cell)
4. Retrieve the data from the papers into the data table and analyse them according to the topic research questions (qualitative synthesis), keep a list of the papers excluded from analysis and criteria applied, write up qualitative analysis. Save an RM database corresponding with box eight of the flow diagram (“…included qualitative”, and one corresponding with the papers excluded, send to EMCDDA
5. Analyse the papers that allow quantitative assessment, keep list of exclusions and criteria used, write up quantitative (meta-)analysis and/or elements (figure, table) Save an RM database corresponding with box nine of the flow diagram (“...included quantitative”), and one with the papers excluded, send to EMCDDA

Table S2. Availability of routine monitoring data on hepatitis C and PWID-related indicators by country

|  | HCV notifications acute PWID 2011 | HCV notifications chronic or unknown PWID 2011 | HCV ab prevalence, 2008-2011 | HCV ab prevalence in new PWID 2008-2011 (incidence proxy) | PWID population size national (2006-2011) | HIV prevalence in PWID 2008-2011 (population level co-infection) | Mortality due to chronic liver disease in the general population | NSP provision quantified | OST provision quantified | HIV ART provision quantified for PWID |
| --- | --- | --- | --- | --- | --- | --- | --- | --- | --- | --- |
| Austria | - | - | yes | yes | - | yes | yes | yes | yes | yes |
| Belgium | - | - | yes | yes | yes | yes | yes | yes | yes | - |
| Bulgaria | - | - | yes | yes | - | yes | yes | yes | yes | yes |
| Cyprus | - | - | yes | yes | yes | yes | yes | yes | yes | - |
| Czech Rep. | - | yes | yes | yes | yes | yes | yes | yes | yes | yes |
| Denmark | yes | yes | yes | - | - | - | yes | - | yes | - |
| Estonia | yes | yes | yes | - | - | yes | yes | yes | yes | yes |
| Finland | - | yes | yes | yes | - | yes | yes | yes | yes | yes |
| France | - | - | - | - | - | yes | yes | yes | yes | - |
| Germany | - | yes | yes | yes | - | yes | yes | yes | yes | yes |
| Greece | - | - | yes | yes | yes | yes | yes | yes | yes | yes |
| Hungary | yes | - | yes | yes | yes | yes | yes | yes | yes | - |
| Ireland | yes | yes | - | - | - | - | yes | - | yes | - |
| Italy | yes | - | yes | - | - | yes | yes | yes | yes | - |
| Latvia | - | yes | yes | - | - | yes | yes | yes | yes | yes |
| Lithuania | yes | - | - | - | - | yes | yes | yes | yes | yes |
| Luxembourg | - | yes | - | - | yes | yes | yes | yes | yes | yes |
| Malta | - | yes | yes | - | - | yes | yes | yes | yes | - |
| Netherlands | yes | - | yes | - | yes | yes | yes | yes | yes | yes |
| Poland | - | - | yes | yes | - | yes | yes | yes | yes | yes |
| Portugal | yes | yes | yes | yes | - | yes | yes | yes | yes | yes |
| Romania | - | yes | yes | yes | - | yes | yes | yes | yes | - |
| Slovakia | yes | yes | yes | yes | yes | yes | yes | yes | yes | yes |
| Slovenia | - | - | yes | yes | - | yes | yes | yes | yes | yes |
| Spain | - | - | - | - | yes | yes | yes | yes | yes | yes |
| Sweden | - | yes | yes | yes | - | yes | yes | yes | yes | - |
| UK | - | yes | yes | yes | yes | yes | yes | - | yes | yes |

Sources:

HCV notifications: ECDC, 2013 (Table A6) [231] http://www.ecdc.europa.eu/en/publications/Publications/Hepatitis-B-C-surveillance-report-2006-2011.pdf

HCV and HIV prevalence: <http://www.emcdda.europa.eu/stats12#inf:displayTables>

PWID population size: http://www.emcdda.europa.eu/stats12#pdu:displayTables

NSP (needle and syringe programmes) and OST (opioid substitution treatment) provision: http://www.emcdda.europa.eu/stats12#hsr:displayTables

Mortality due to chronic liver disease: Eurostat, <http://epp.eurostat.ec.europa.eu/tgm/table.do?tab=table&plugin=1&language=en&pcode=tps00131>

HIV ART provision: Mathers et al. Lancet, 2010 [232]

1. [↑](#footnote-ref-1)
