# Supplementary material for: Hepatitis C Virus Infection Epidemiology among People Who Inject Drugs in Europe: A Systematic Review of Data for Scaling Up Treatment and Prevention
Source: PLoS One. 2014 Jul 28;9(7):e103345. doi: 10.1371/journal.pone.0103345 (PMC4113410; doi:10.1371/journal.pone.0103345)

Web-appendix S3: Additional detail on Chronicity analyses

***Methodology***

The pooled chronicity rate was calculated as the average, weighted by the number of injecting drug users that were included in each result. Pooled odds ratio’s (OR) were estimated assuming a random-effects model.

Random-effects meta-regression and stratification was used to formally compare differences in chronicity rate estimates across study characteristic categories with the among-study variance estimated by restricted maximum likelihood. Stratified summary estimates allowed descriptive comparisons across individual categories of study characteristics (i.e., summary estimates and 95% confidence intervals for each category). Variation between estimates was evaluated by comparing Cochran’s Q two-sided p value with a 0.05 significance level.

For these analyses, at least three study estimates in each stratum were required.

Meta-regression analysis was conducted in Comprehensive Meta Analysis version 2.

***
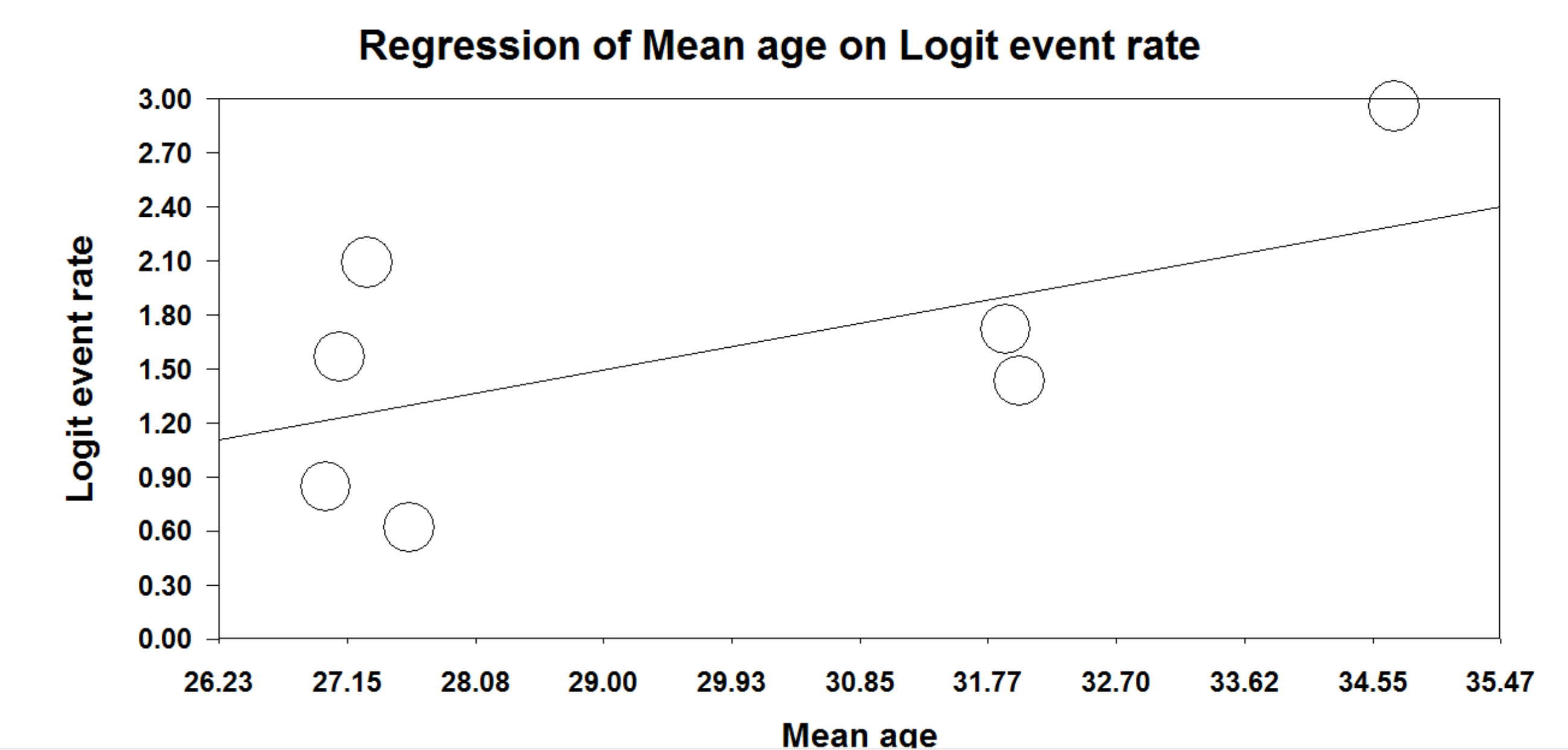
*** ***Relationship between mean age of population and chronicity rate***

**
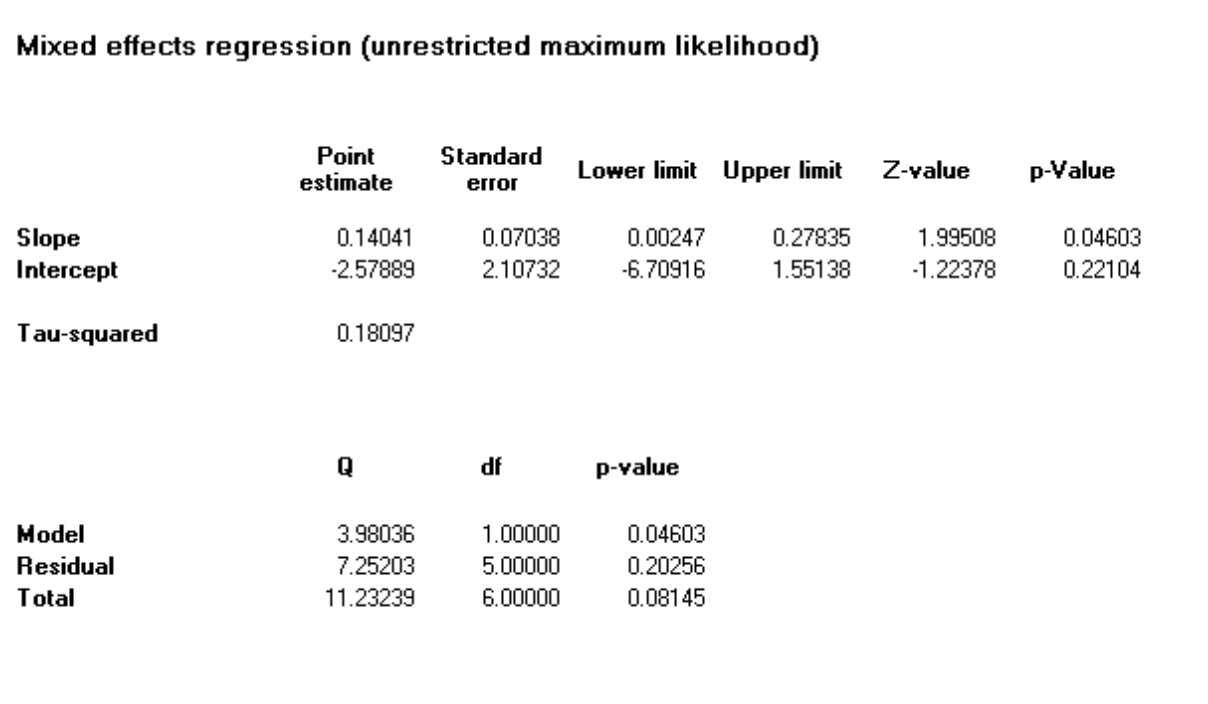
**

**
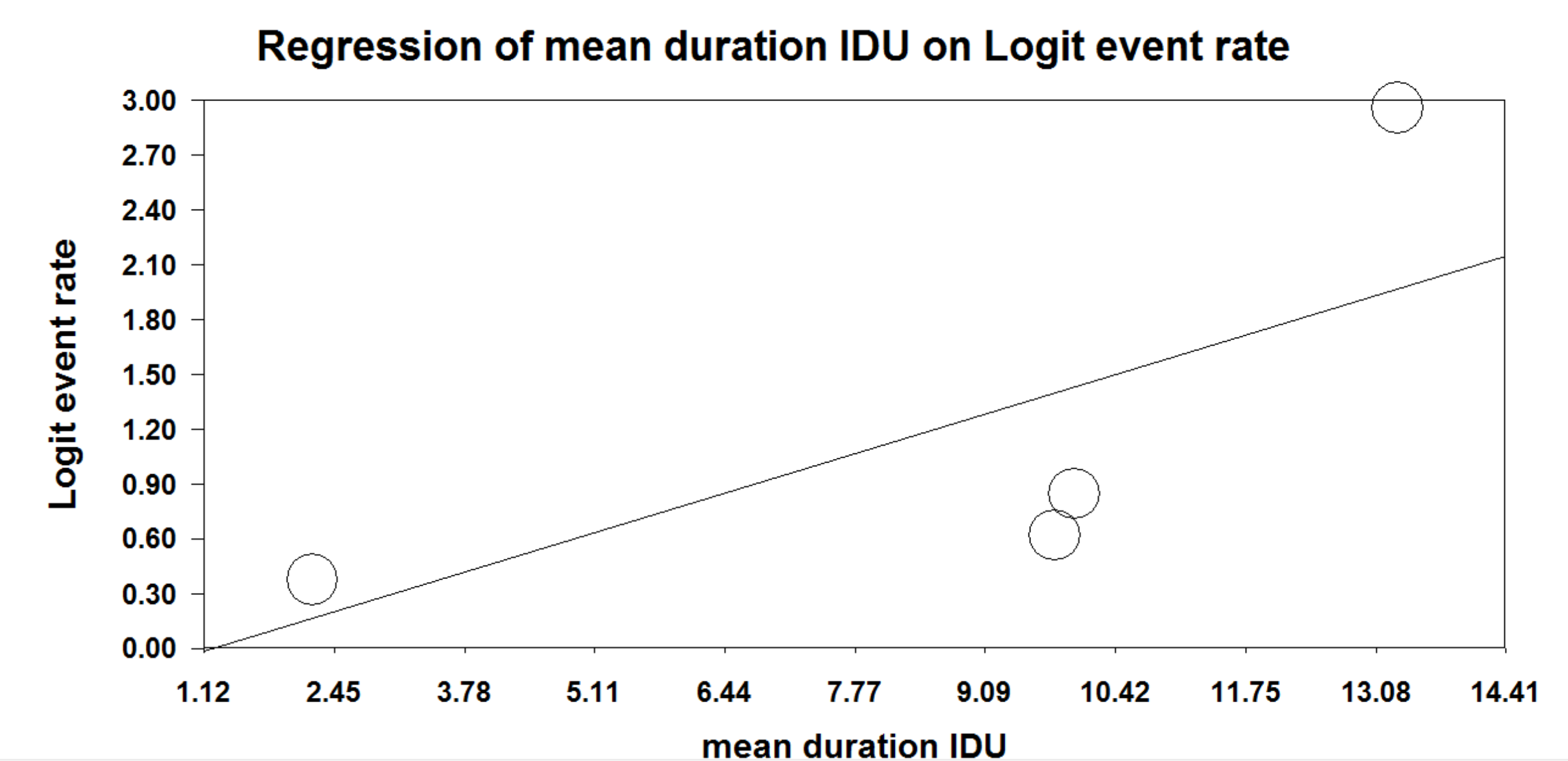
*Relationship between mean duration of injecting drug use of population and chronicity rate***

**
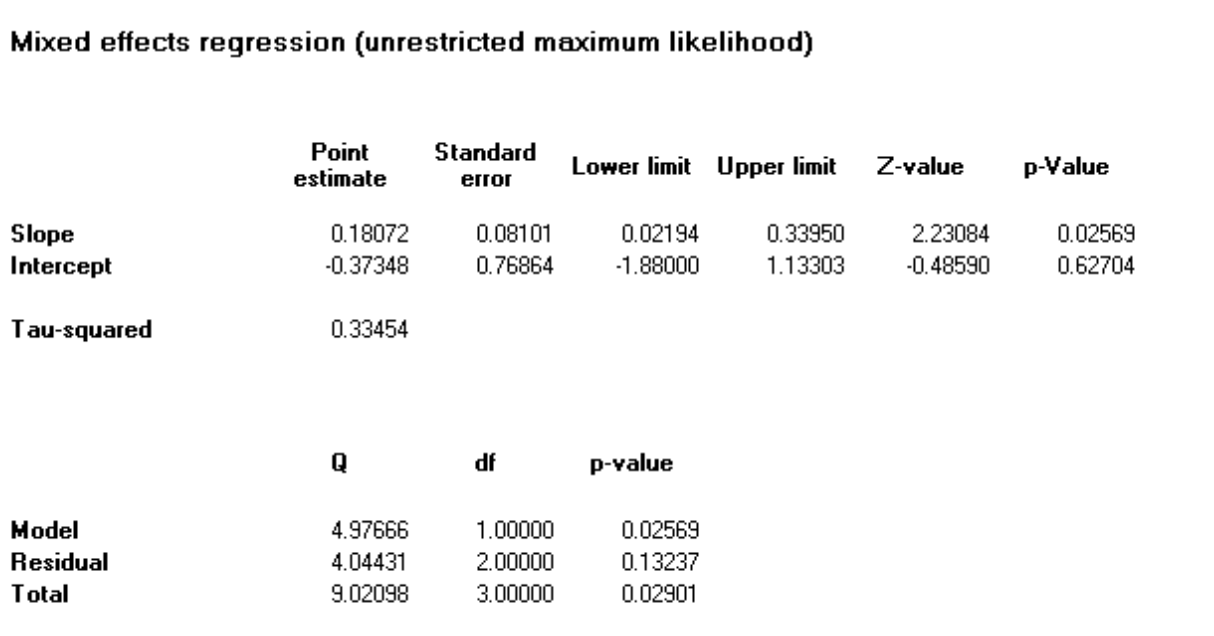
**

***Relation gender and chronicity rate***


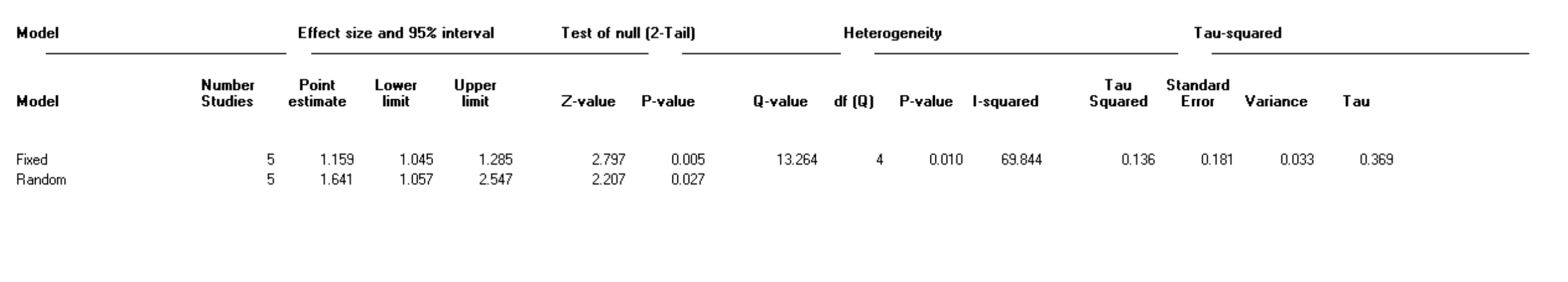


***HIV co-infection and chronicity rate***


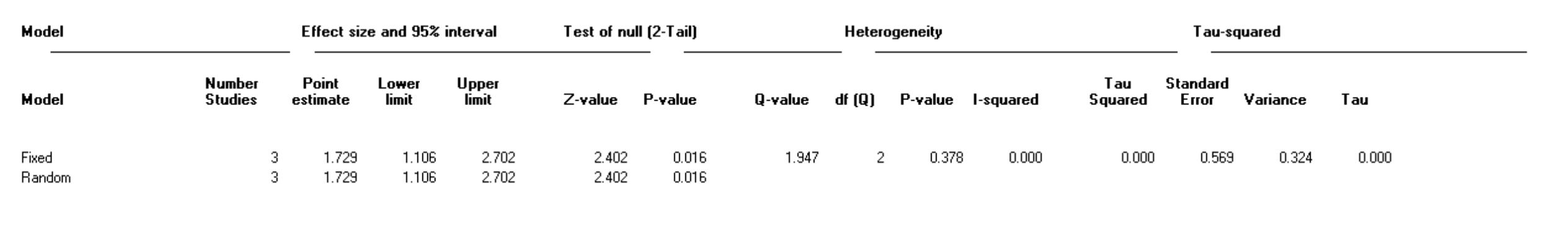


***HBV co-infection and chronicity rate***


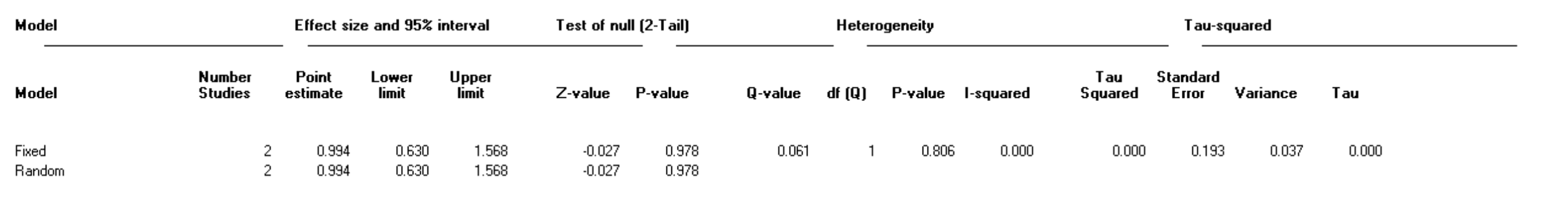


***Effect of setting on chronicity rate***


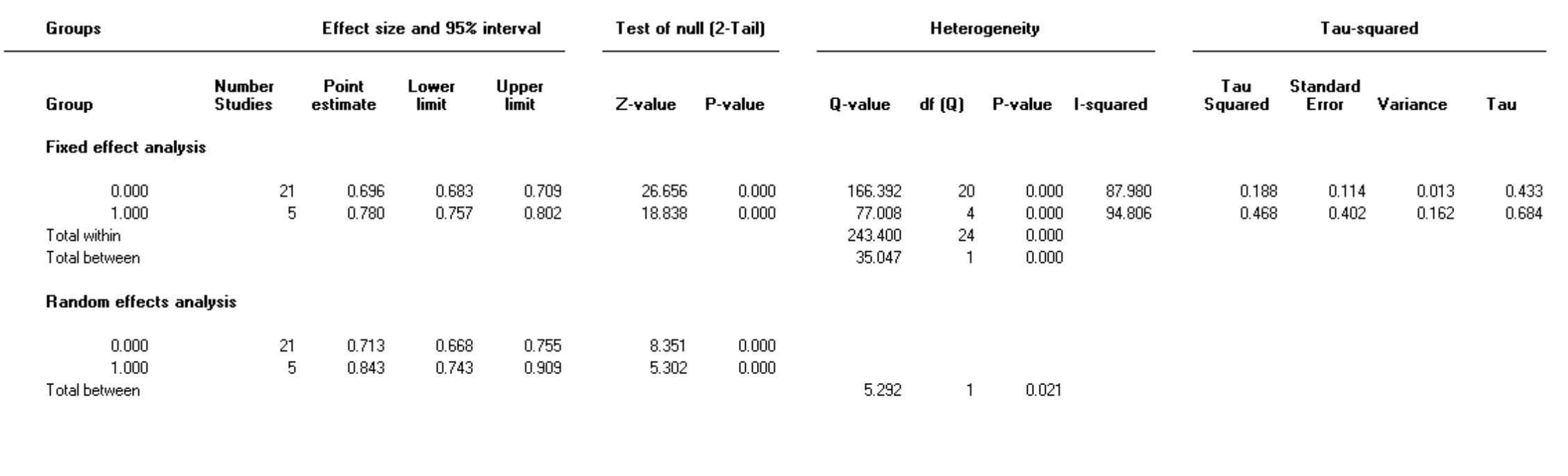

Supplement: Web-appendix S3 — Additional detail on the Chronicity analyses. (DOCX) [file pone.0103345.s003.docx]
